# Effects of intensive blood pressure treatment on orthostatic hypertension: individual level meta-analysis

**DOI:** 10.1136/bmj-2024-080507

**Published:** 2025-03-25

**Authors:** Stephen P Juraschek, Jiun-Ruey Hu, Jennifer L Cluett, Carol Mita, Lewis A Lipsitz, Lawrence J Appel, Nigel S Beckett, Barry R Davis, Rury R Holman, Edgar R Miller, Kenneth J Mukamal, Ruth Peters, Jan A Staessen, Addison A Taylor, Jackson T Wright, William C Cushman

**Affiliations:** 1 Beth Israel Deaconess Medical Center, Harvard Medical School, Boston, MA, USA; 2 Department of Cardiology, Smidt Heart Institute, Cedars-Sinai Medical Center, Los Angeles, CA, USA; 3 Countway Library, Harvard University, Boston, MA, USA; 4 Hebrew SeniorLife, Hinda and Arthur Marcus Institute for Aging Research and Harvard Medical School, Boston, MA, USA; 5 Johns Hopkins University, Baltimore, MA, USA; 6 Department of Ageing and Health, Guy's and St Thomas' NHS Foundation Trust, London, UK; 7 Department of Biostatistics and Data Science, Coordinating Center for Clinical Trials, The University of Texas School of Public Health, Houston, TX, USA; 8 Diabetes Trials Unit, Radcliffe Department of Medicine, University of Oxford, Oxford, UK; 9 The George Institute for Global Health, Sydney, NSW, Australia; 10 The School of Population Health, University of New South Wales, Sydney, NSW, Australia; 11 Alliance for the Promotion of Preventive Medicine (APPREMED), Mechelen, Belgium; 12 Department of Cardiovascular Medicine, Shanghai Key Laboratory of Hypertension, Shanghai Institute of Hypertension, State Key Laboratory of Medical Genomics, National Research Centre for Translational Medicine, Ruijin Hospital, Shanghai Jiaotong University School of Medicine, Shanghai, China; 13 Biomedical Research Group, Faculty of Medicine, University of Leuven, Leuven, Belgium; 14 Michael E DeBakey VA Medical Center and Baylor College of Medicine, Houston, TX, USA; 15 Case Western Reserve University, University Hospitals Cleveland Medical Center, Cleveland, OH, USA; 16 University of Tennessee Health Science Center, Memphis, TN, USA

## Abstract

**Objective:**

To determine the effects of intensive blood pressure treatment on orthostatic hypertension.

**Design:**

Systematic review and individual participant data meta-analysis.

**Data sources:**

MEDLINE, Embase, and Cochrane CENTRAL databases through 13 November 2023.

**Inclusion criteria:**

Population: ≥500 adults, age ≥18 years with hypertension or elevated blood pressure; intervention: randomized trials of more intensive antihypertensive drug treatment (lower blood pressure goal or active agent) with duration ≥6 months; control: less intensive antihypertensive drug treatment (higher blood pressure goal or placebo); outcome: measured standing blood pressure.

**Main outcomes:**

Orthostatic hypertension, defined as an increase in systolic blood pressure ≥20 mm Hg or diastolic blood pressure ≥10 mm Hg after changing from sitting to standing.

**Data synthesis:**

Two investigators independently abstracted articles. Individual participant data from nine trials identified during the systematic review were appended together as a single dataset.

**Results:**

Of 31 124 participants with 315 497 standing blood pressure assessments, 9% had orthostatic hypotension (that is, a drop in blood pressure after standing of systolic ≥20 mm Hg or diastolic ≥10 mm Hg), 17% had orthostatic hypertension, and 3.2% had both a rise in systolic blood pressure and standing blood pressure ≥140 mm Hg at baseline. The effects of more intensive treatment were similar across trials with odds ratios for orthostatic hypertension ranging from 0.85 to 1.08 (I^2^=38.0%). During follow-up, 17% of patients assigned to more intensive treatment had orthostatic hypertension, whereas 19% of those assigned less intensive treatment had orthostatic hypertension. Compared with less intensive treatment, the risk of orthostatic hypertension was lower with more intensive blood pressure treatment (odds ratio 0.93, 95% confidence interval 0.90 to 0.96). Effects were greater among non-black versus black adults (odds ratio 0.86 *v* 0.97; P for interaction=0.003) and adults without diabetes versus those with diabetes (0.88 *v* 0.96; P for interaction=0.05) but did not differ by age ≥75 years, sex, baseline seated blood pressure ≥130/≥80 mm Hg, obesity, stage 3 kidney disease, stroke, cardiovascular disease, standing systolic blood pressure ≥140 mm Hg, or pre-randomization orthostatic hypertension (P for interactions ≥0.05).

**Conclusions:**

In this pooled cohort of adults with elevated blood pressure or hypertension, orthostatic hypertension was common and more intensive blood pressure treatment modestly reduced the occurrence of orthostatic hypertension. These findings suggest that approaches generally used for seated hypertension may also prevent hypertension on standing.

**Study registration:**

Prospero CRD42020153753 (original proposal).

## Introduction

Orthostatic hypertension, an elevation in blood pressure after standing, is an emerging risk factor for several adverse health outcomes, including cardiovascular disease, stroke, kidney disease, and cognitive impairment.^1^ ^2^ ^3^ Orthostatic hypertension also seems to be an important predictor of all cause mortality among older adults.^4^ Although individual cohort studies have observed that orthostatic hypertension disproportionately affects adults with hypertension, the effects of blood pressure treatment on the occurrence of orthostatic hypertension have not been systematically examined.

In a recent, individual participant data meta-analysis of hypertension trials with standing blood pressure assessments, we examined the effect of more intensive blood pressure treatment on orthostatic hypotension.^5^ However, we did not examine the effect of treatment on orthostatic hypertension, which was also collected during these trials. Current recommendations for the treatment of orthostatic hypertension focus on agents that are not considered first line for seated hypertension (that is, thiazide diuretics, dihydropyridine calcium channel blockers, or angiotensin converting enzyme inhibitors/angiotensin receptor blockers).^6^ Whether more intensive treatments generally used for seated hypertension might be efficacious for orthostatic hypertension is unknown, but it could have implications for the formulation of treatment strategies to tackle this emerging hypertensive phenotype.

The objectives of this study were to use the individual participant data combined from the nine hypertension trials identified by the systematic review above to determine the prevalence of orthostatic hypertension among adult participants of hypertension treatment trials and the effect of more intensive blood pressure treatment (that is, a lower blood pressure treatment goal or active therapy versus either a higher blood pressure treatment goal or placebo) on orthostatic hypertension and to assess for effect modification by demographic characteristics.

## Methods

### Search strategy and eligibility criteria

This post hoc study focuses on orthostatic hypertension, but the search strategy of our original systematic review, focused on orthostatic hypotension, was described elsewhere.^5^ ^7^ In brief, our review was registered in the PROSPERO registry on 28 April 2020 (CRD42020153753) and initial searches included MEDLINE/PubMed), Embase, and the Cochrane Central Register of Controlled Trials databases without language restrictions. A research librarian (CM) prepared our search strategy, which focused on hypertension, blood pressure treatment, standing blood pressure (particularly orthostatic hypotension), and randomized trials. Duplicate records were removed in EndNote, and two independent investigators (SPJ and JLC) screened abstracts with Covidence, with discrepancies adjudicated by consensus. This search was updated to include citations through 13 November 2023 (supplementary methods 1; supplementary figure A). The search ultimately entailed 1127 unique abstracts and 70 unique trials. Only one of the eligible trials was excluded owing to inability to share individual participant data. Because our original search included all trials with standing blood pressure measurements, we were able to use the outcomes of this search to examine orthostatic hypertension as a post hoc analysis.

The original systematic review was guided by the following PICO (population, intervention, comparison, outcomes) criteria.^8^ Population: trials of at least 500 adults (ages 18 years or older) with elevated blood pressure or hypertension (based on seated measurements). Intervention: at least six months of randomized antihypertensive drug treatment (blood pressure goal or active agent). Comparison: at least two blood pressure goals (one less than the other) or active therapy versus placebo. Outcome: orthostatic hypotension measured after randomization. Although orthostatic hypotension was the outcome of the original search, all these trials also had the relevant data for calculation of orthostatic hypertension. We excluded trials of pregnant women or children, animal experiments (non-human trials), reviews, observational studies, and studies without direct measures of orthostatic hypotension (for example, based on self-report or claims). We pooled trials together overall and by type—that is, those comparing two treatment goals (a lower versus a higher goal) or placebo controlled trials.

In addition to our systematic review above, we also attempted to contact investigators of trials of antihypertensive drug treatment in adults with elevated blood pressure or hypertension included in a recent meta-analysis focused on cardiovascular disease,^9^ asking about the availability of standing blood pressure measurements. This process led to the inclusion of one trial not identified through our original search.^10^ One trial was not able to provide us with data owing to data sharing restrictions.^11^ All trials identified had both pre-randomization and post-randomization orthostatic blood pressure assessments, which could be used to derive orthostatic hypertension. Risk of bias characterization was updated to reflect orthostatic hypertension as the primary outcome of this systematic review (supplementary table A).^12^

### Treatment assignment

Similarly to our previous work, we chose a priori to examine pooled effects by categories of trial design: trials of blood pressure treatment goal (that is, one goal lower than the other goal) and trials of an active antihypertensive agent versus placebo. More intensive treatment included patients assigned a lower blood pressure treatment goal and those assigned to active antihypertensive treatment, and less intensive treatment included those assigned a higher blood pressure treatment goal and those assigned to placebo.

### Orthostatic hypertension

We determined the difference between standing minus seated blood pressure for each trial at all available visits (a visit being a clinical session whereby a participant interacted with a study team and blood pressure was measured). We defined orthostatic hypertension as standing minus seated systolic blood pressure of ≥20 mm Hg or diastolic blood pressure of ≥10 mm Hg, the definition used in SPRINT and our previous work.^13^ ^14^ ^15^ ^16^ ^17^ Seated blood pressure varied by trial protocol—for example, based on one measurement or based on the average of two or three measurements (sometimes with the first measurement excluded). Standing blood pressure similarly varied according to trial protocols but often included only a single measurement (see table 1). We defined standing systolic hypertension as a standing systolic blood pressure ≥140 mm Hg. This was incorporated into a recently updated definition of orthostatic hypertension—that is, a change in systolic blood pressure of ≥20 mm Hg and a standing systolic blood pressure of ≥140 mm Hg (the new consensus definition).^18^ ^19^ Orthostatic hypotension was defined as standing minus seated systolic blood pressure of ≤−20 mm Hg or diastolic blood pressure of ≤−10 mm Hg.^20^ Baseline orthostatic hypertension or standing systolic hypertension was based on the seated and standing blood pressures measured in the visit in closest proximity and before or during the randomization visit.

**Table 1:** Characteristics of included trials

Trial name	No of participants	Population characteristics	Standard BP treatment (goal or placebo) or intensive BP treatment (goal or agent), mm Hg	Length of follow-up (years)—median, mean (SD), or range	Antihypertensive agents for intervention	Blood pressure device	Seated BP measurement	Standing BP measurement
**Blood pressure treatment goal trials**								
AASK	1094	18-70 year old African American patients with hypertensive renal disease, without diabetes; seated DBP ≤95 mm Hg	MAP 102-107 or MAP ≤92	3-6.4	First line: 1 of 3 agents—metoprolol (50-200 mg/d), ramipril (2.5-10 mg/d), or amlodipine (5-10 mg/d)	Hawksley Random Zero	Mean of last 2 of 3 measures	1 measure after 2:45 min of standing
ACCORD BP	4733	≥40 year old patients with diabetes and CV disease or ≥55 year old patients with diabetes with CV risk factors; seated SBP 130-180 mmHg*	SBP <140 or SBP <120	4.7	First line: combination of diuretic and either ACE inhibitor or β blocker	Omron HEM-907	Mean of 3 measures	Mean of 3 measures, 1 min after standing, each measure separated by 1 min
SPRINT	9361	≥50 year old patients at high risk for CV disease but who do not have stroke or DM; seated SBP 130-180 mm Hg* and standing SBP <110 mm Hg	SBP <140 or SBP <120	3.3	First line: thiazide-type diuretic encouraged, loop diuretics (advanced chronic kidney disease), and β adrenergic blockers (coronary artery disease); chlorthalidone was encouraged as primary thiazide-type diuretic and amlodipine as preferred calcium channel blocker	Omron HEM-907	Mean of 3 measures	1 measure, 1 min after standing
SPS3	3020	≥30 year old patients who had recent lacunar stroke; seated SBP ≥140 mm Hg or seated DBP ≥90 mm Hg and diagnosis of hypertension	SBP 130-149 or SBP <130	3.7 (SD 2.0)	Clinician directed antihypertensive regimen	Colin Press-Mate BP-8800C	Mean of 3 measures	1 measure, 2 min after standing
UKPDS	1148	25-65 year old patients with diabetes and hypertension; seated SBP ≥160 mm Hg or seated DBP ≥90 mm Hg (≥150/≥85 if on antihypertensive drugs)	BP <180/105 or BP <150/85	8.4	First line: captopril (25 mg/d to 50 mg bid) or atenolol (50-100 mg/d)	Copal UA-251, Takeda UA-751, or Hawksley Random Zero	Mean of last 3 of 4 measures†	1 measure, 1 min after standing
**Placebo controlled trials**								
HYVET	3845	≥80 year old patients with hypertension; seated SBP 160-199 mm Hg, standing SBP ≥140 mm Hg, seated DBP 90-109 mm Hg‡	Placebo or <150/ <80	1.8	First line: indapamide (sustained release, 1.5 mg) or matching placebo alone	Mercury sphygmomanometer or validated automated device	Mean of 2 measures	Mean of 2 measures after 2 min of standing
SHEP	4736	≥60 year old patients with isolated systolic hypertension; seated SBP 160-219 mm Hg§, standing SBP ≥140 mm Hg, seated DBP <90 mm Hg	Placebo or SBP <160 if baseline SBP was >180; 20 mm Hg reduction if baseline SBP was 160-179	4	Step 1: chlorthalidone 12.5-25 mg/d; step 2: atenolol 25-50 mg/d (or reserpine 0.05-0.1 mg/d)	Hawksley random zero	Mean of 2 measures	2 measurements, after 1 and 3 min of standing
SYST-EUR	4695	≥60 year old patients with isolated systolic hypertension; seated SBP <220 mm Hg, standing SBP ≥140 mm Hg, seated DBP <95 mm Hg	Placebo or SBP <150 (reduction of ≥20 mm Hg)	2	Nitrendipine (10 mg/day to 20 mg bid) combined with or replaced by enalapril (5 mg/d to 20 mg/d), hydrochlorothiazide (12.5-25 mg/d), or both. Goal to reduce sitting SBP by ≥20 mm Hg to <150 mm Hg. Placebos were identical to study drugs, with similar schedule	Unspecified, conventional sphygmomanometers	Mean of 2 measures	2 measures after 2 min of standing
TOMHS	902	45-69 year old patients with mild hypertension; DBP 90-99 mm Hg	Nutritional-hygienic intervention + placebo or nutritional-hygienic intervention + 1 of 5 arms¶: acebutalol, amlodipine, chlorthalidone, doxazosin, or enalapril	4.4	Chlorthalidone 15-30 mg/d, acebutalol, 400-800 mg/d, doxazosin mesylate 2-4 mg/d, amlodipine maleate 5-10 mg/d, or enalapril maleate 5-10 mg/d. Doses were doubled or chlorthalidone or enalapril was added if DBP was ≥95 mm Hg (3 successive visits or ≥105 mm Hg during single visit). Participants assigned to placebo group were given chlorthalidone if BP was not controlled with nutritional-hygienic intervention alone	Random-zero sphygmomanometer	Mean of 2 measures	1 measure, 2 min after standing

Adapted from Juraschek et al, *Annals of Internal Medicine*, 2021.^5^

ACE=angiotensin converting enzyme; bid=twice daily; BP=blood pressure; CV=cardiovascular; DBP=diastolic blood pressure; DM=diabetes mellitus; MAP=mean arterial pressure; SBP=systolic blood pressure; SD=standard deviation.

* Range varied by baseline antihypertensive use.

† Of 4 measures, first was discarded and mean of next 3 consecutive readings (with coefficient of variation <15%) was used.

‡ Average seated DBP was later changed to <110 mm Hg.

§ Range was 130-219 mm Hg and DBP <85 mm Hg if prescribed antihypertensive agents.

¶ These arms were combined to represent “intensive blood pressure treatment group” in this meta-analysis.

### Other covariates

We obtained the following covariate information from each trial: age, sex (women, men), race (black, non-black; this was not universally available), pre-randomization seated and standing systolic and diastolic blood pressure, baseline creatinine or estimated glomerular filtration rate or chronic kidney disease status, body mass index, diabetes status, previous stroke, and history of cardiovascular disease. We defined obesity as body mass index ≥30 and stage 3 chronic kidney disease as estimated glomerular filtration rate <60 mL/min/1.73 m^2^ on the basis of the 2021 CKD-EPI race-free, creatinine equation^21^ or self-reported history of kidney disease (SHEP trial only). Differences in the definitions of diabetes, stroke, and cardiovascular disease between studies were described elsewhere.^5^ ^7^

### Statistical analysis

Pre-randomization and post-randomization visit data from all trials were appended into a single analytic dataset before pooled analyses. Analyses were restricted to blood pressure, body mass index, and estimated glomerular filtration rate measures between the 0.01st and 99.99th centiles of all measurements (baseline and follow-up) from all nine trials to account for biologically implausible outliers (particularly relevant for stratified analyses; see supplementary table B for values corresponding to these thresholds and supplementary table C for values corresponding to 0.1st and 99.9th centiles). We summarized population characteristics via means and proportions overall, by orthostatic hypertension status, by trial type, and according to each study. We used kernel density plots (bandwidth 5) to visually examine the distribution of systolic and diastolic blood pressure in seated and standing positions and the difference between positions (standing minus seated) according to the pre-randomization visit and follow-up visits among participants assigned to a lower blood pressure treatment goal or active therapy and among those assigned to a higher blood pressure treatment goal or placebo across all studies. We compared characteristics between participants with and without a baseline orthostatic hypertension assessment.

We plotted the proportion of orthostatic hypertension detected during study visits grouped according to a series of time intervals: month 0/pre-randomization, after randomization to ≤1 month, >1 to ≤6 months, >6 to ≤12 months, >12 to ≤24 months, >24 to ≤36 months, >36 to ≤48 months, and >48 months. We plotted proportions overall according to assignment (that is, a lower treatment goal/active therapy or a higher treatment goal/placebo) via generalized estimating equations (Poisson family, log link, robust variance estimator, exchangeable correlation matrix) without adjustment. We tabulated the number of measurements and number of individual participants at risk, determining the proportion with orthostatic hypertension at any time during each time period. We also examined the proportions, changes in proportion, and odds of orthostatic hypertension over time, using generalized estimating equations (binomial family, logit link, robust variance estimator, exchangeable correlation matrix) adjusted for study. We used generalized estimating equations to account for repeated measurements within participants as they are able to generate valid variance estimates even when the within group correlation structure is mis-specified. Models included interaction terms to assess for differences at different time points. We also examined the relation between baseline orthostatic hypertension and follow-up orthostatic hypertension via generalized estimating equations (binomial family, logit link, robust variance estimator, exchangeable correlation matrix), using an interaction term with randomized treatment assignment to assess whether this relation differed by treatment.

In addition, we compared the effect of more intensive treatment (that is, a lower treatment goal or active therapy) versus higher treatment goals or placebo on the odds of orthostatic hypertension during follow-up visits, using generalized estimating equations (binomial family, logit link, robust variance estimator, exchangeable correlation matrix). We did these analyses for individual trials and pooled by trial type (that is, the five blood pressure treatment goal trials and the four placebo controlled trials) and overall. We repeated this as a sensitivity analysis using a Poisson family log link.

We repeated models using alternate definitions of orthostatic hypertension (described above) both for 0.01st to 99.99th centiles of blood pressure values and with truncation at 0.1st and 99.9th centiles. We also determined mean systolic or diastolic blood pressure before and after randomization and treated orthostatic change in systolic or diastolic blood pressure as a continuous outcome (these models used a normal family, identity link; by contrast, all models with alternate definitions of orthostatic hypertension as dichotomous outcome variables used a binomial family logit link).

Moreover, we did subgroup analyses examining orthostatic hypertension in the following pre-specified strata: age (≤75 or >75 years), sex (men or women), race (non-black or black), pre-randomization seated blood pressure (systolic blood pressure ≥130 mm Hg or diastolic blood pressure ≥80 mm Hg, no or yes), diabetes (no or yes), previous stroke (no or yes), stage 3 chronic kidney disease (<60 or ≥60 mL/min per 1.73 m^2^; in SHEP, kidney disease was self-reported), body mass index (<30 or ≥30), history of cardiovascular disease (no or yes), standing systolic blood pressure before randomization (<140 or ≥140 mm Hg), and pre-randomization orthostatic hypertension (no or yes). We used interaction terms to compare effects across strata. We repeated these analyses using the recent consensus definition for orthostatic hypertension,^18^ ^19^ as well as with truncation at the 0.1st and 99.9th centiles of continuous covariates.

We did all analyses for the nine trials as well as by trial type—that is, the five trials that compared two blood pressure treatment goals or the four placebo controlled trials. We used a two stage meta-analysis with a random effects model weighted by the inverse variance in sensitivity analyses and evaluated heterogeneity between studies via the I^2^ statistic.^22^ We examined heterogeneity by trial design and overall. Although our a priori intention was to pool studies, this plan was subject to evaluation of heterogeneity (both its magnitude and direction of effect).^23^ Small study effects were assessed via Egger’s test and funnel plots.^24^ We used Stata 15.1 for all statistical analyses. We considered a two tailed P value of <0.05 without adjustment for multiple comparisons to be statistically significant. A dummy dataset and analytic codes are available at https://doi.org/10.7910/DVN/RHUF9F; the code is also available in supplementary methods 2.

### Patient and public involvement

The original systematic review was initiated without patient or public involvement. However, a patient of SPJ with a history of orthostatic hypertension was a motivation for this work and reviewed this manuscript at the time of the revision request. This patient’s feedback was incorporated into the manuscript.

## Results

### Population characteristics

Of the 31 124 participants with 315 497 measurements contributing to this individual participant data meta-analysis, the mean age was 67.6 (standard deviation (SD) 10.4) years with 25.1% over the age of 75 years; 47.4% of participants were women (table 2). Differences between participants with and without orthostatic assessments at baseline are found in supplementary table D. Before randomization, the mean seated systolic blood pressure was 152.6 (SD 21.3) mm Hg and the mean seated diastolic blood pressure was 80.9 (11.5) mm Hg. After standing, systolic blood pressure was 152.3 (SD 21.2) mm Hg and diastolic blood pressure was 83.9 (12.1) mm Hg, with a mean postural change in systolic blood pressure of −1.9 (SD 11.4) mm Hg and in diastolic blood pressure of 2.4 (7.4) mm Hg (see supplementary table E for similar results based on all available pre-randomization visits). Before randomization, 8.7% of participants had orthostatic hypotension, 16.7% had orthostatic hypertension, and 1.9% had standing hypertension. The distribution of systolic and diastolic blood pressure became narrower with treatment and shifted to the left (supplementary figures B and C). Little change occurred in the distribution of orthostatic changes before and after treatment, regardless of assignment.

**Table 2:** Participants’ characteristics

Characteristic	All trials			No orthostatic hypertension at baseline			Orthostatic hypertension at baseline			Treatment goal trials			Placebo controlled trials	
	No	Mean (SD) or %		No	Mean (SD) or %		No	Mean (SD) or %		No	Mean (SD) or %		No	Mean (SD) or %
Age, years	31 120	67.6 (10.4)		22 743	68.5 (10.7)		4567	67.3 (10.1)		18 547	64.5 (9.9)		12 573	72.3 (9.2)
Age >75 years	31 120	25.1		22 743	28.4		4567	23.0		18 547	15.7		12 573	39.1
Women	31 124	47.4		22 745	47.7		4569	47.6		18 547	38.9		12 577	59.9
Black	24 125	26.1		16 340	25.2		3978	32.4		18 547	29.5		5578	14.7
Seated SBP*, mm Hg	30 988	152.6 (21.3)		22 745	155.2 (21.2)		4569	149.0 (21.7)		18 512	141.3 (17.6)		12 476	169.4 (14.2)
Standing SBP*, mm Hg	27 353	152.3 (21.2)		22 745	151.3 (21.0)		4569	157.1 (21.1)		14 877	142.1 (19.7)		12 476	164.5 (15.7)
Postural change in SBP*, mm Hg	27 346	−1.9 (11.4)		22 745	−3.9 (9.9)		4569	8.1 (13.0)		14 870	0.6 (12.3)		12 476	−4.9 (9.4)
Seated DBP*, mm Hg	30 970	80.9 (11.5)		22 745	82.4 (11.2)		4569	77.2 (12.0)		18 518	79.1 (12.2)		12 452	83.7 (9.8)
Standing DBP*, mm Hg	27 338	83.9 (12.1)		22 745	82.6 (11.5)		4569	90.6 (12.1)		14 879	83.0 (13.4)		12 459	85.1 (10.1)
Postural change in DBP*, mm Hg	27 326	2.4 (7.4)		22 745	0.2 (5.5)		4569	13.4 (6.0)		14 878	3.2 (7.8)		12 448	1.4 (6.8)
eGFR†, mL/min/1.73 m^2^	24 939	72.1 (20.1)		17 801	69.8 (19.7)		3539	72.7 (20.2)		17 050	74.8 (21.4)		7889	66.4 (15.4)
Stage III CKD†	30 542	28.1		22 339	30.8		4469	26.9		18 095	25.1		12 447	32.4
Body mass index	30 940	28.9 (5.6)		22 611	28.4 (5.3)		4538	29.4 (6.1)		18 453	30.3 (5.8)		12 487	27.0 (4.5)
Obesity	30 940	35.8		22 611	32.0		4538	38.5		18 453	46.0		12 487	20.9
Diabetes	31 121	24.7		22 743	16.5		4569	15.6		18 546	34.9		12 575	9.6
Previous stroke	25 833	12.9		20 887	9.9		4123	14.6		13 258	22.8		12 575	2.4
History of CVD	30 021	14.9		21 876	12.7		4343	13.8		17 457	20.4		12 564	7.3
Standing SBP ≥140 mm Hg*	27 353	1.9		22 745	2.1		4569	0.7		14 877	3.4		12 476	0.1
Orthostatic hypotension‡	27 314	8.7		22 745	10.1		4569	2.0		14 866	8.5		12 448	9.0
Orthostatic hypertension§	27 314	16.7		22 745	0.0		4569	100		14 866	21.0		12 448	11.6
Orthostatic hypertension (consensus definition)§	27 314	3.2		22 745	0.0		4569	100		14 866	5.1		12 448	0.8

Some covariates were missing at baseline. These participants were not excluded if they were randomized and had follow-up orthostatic hypertension assessments.

CKD=chronic kidney disease; CVD=cardiovascular disease; DBP=diastolic blood pressure; eGFR=estimated glomerular filtration rate; SBP=systolic blood pressure; SD=standard deviation.

* Pre-randomization measurements.

† Based on Chronic Kidney Disease-Epidemiology (CKD-EPI) 2021 race-free, creatinine equation. eGFR was not available from UKPDS or SHEP. Although UKPDS provided stage III CKD categories based on 2021 CKD-EPI equation, self-reported history of kidney disease was relied on for SHEP.

‡ As orthostatic hypertension and orthostatic hypotension are defined on basis of criterion from either systolic or diastolic blood pressure, both definitions can be met at same time (although this is rare).

**Fig 1:**
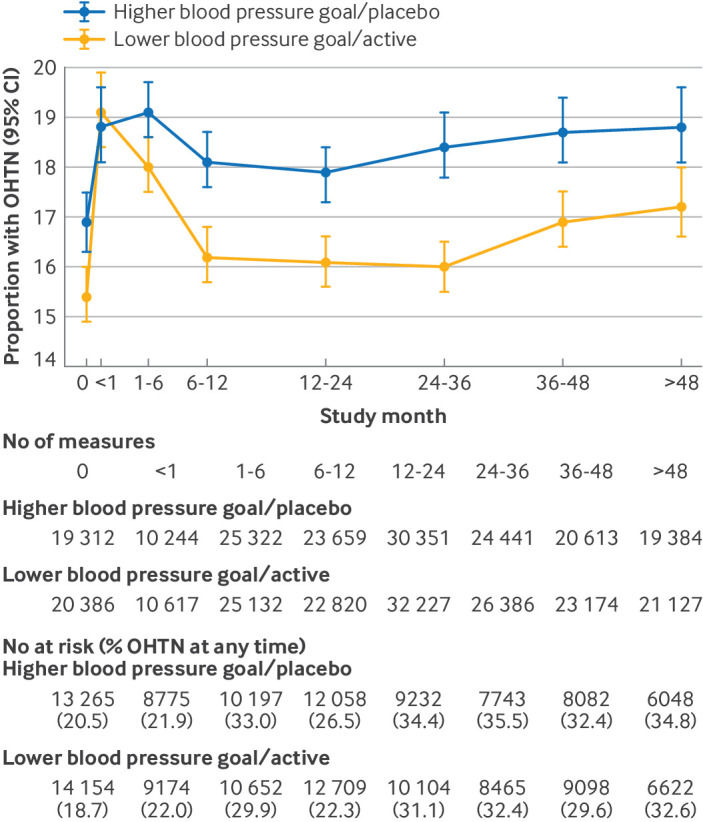
Proportion of participants with orthostatic hypertension by study month. Follow-up visits were grouped together (month 0/before randomization, <1 month, 1-6 months, 6-12 months, 12-24 months, 24-36 months, 36-48 months, and >48 months). Proportions were estimated with generalized estimating equations, using unadjusted Poisson family, log link. This model accounts for correlated within person measurements. Numbers below figure represent measurements contributing to each proportion by time period. Note that some trials had multiple visits before randomization, which contributed to these models and may account for differences in proportion of orthostatic hypertension estimated by this approach, versus descriptive estimate based on single visit in table 2. In addition, number of unique participants at risk in each time period is reported, with percentage with orthostatic hypertension at any time during this time period. These proportions differ from those in figure. BP=blood pressure; CI=confidence interval; OHTN=orthostatic hypertension

§ This table is based on a single visit (visit closest to and preceding randomization). However, some trials had multiple pre-randomization measurements, which were included in models elsewhere (eg, fig 1 and supplementary table E). Consequently, proportion with pre-randomization orthostatic hypertension differs slightly on basis of these two approaches. Orthostatic hypertension was defined as orthostatic increase in SBP ≥20 mm Hg or DBP ≥10 mm Hg. Consensus orthostatic hypertension definition was based on orthostatic increase in SBP ≥20 mm Hg and standing SBP ≥140 mm Hg.

### Proportion of orthostatic hypertension over time

The proportion of participants with orthostatic hypertension increased initially but then decreased over time in both arms, with a greater reduction in the more intensive treatment group (fig 1). We observed a similar pattern with respect to the relative odds of orthostatic hypertension in that a significant increase occurred within the first month in the lower goal/active therapy group compared with the higher goal/placebo group (supplementary table F). However, these initial increases did not persist over time in the lower goal/active therapy group, whereas the odds in the standard group remained elevated compared with pre-randomization. Having orthostatic hypertension before randomization was associated with having orthostatic hypertension during follow-up (odds ratio 2.53, 95% confidence interval (CI) 2.39 to 2.68), and this relation did not differ by randomized treatment assignment (P for interaction=0.61).

### Aggregate effects on orthostatic hypertension

**Fig 2:**
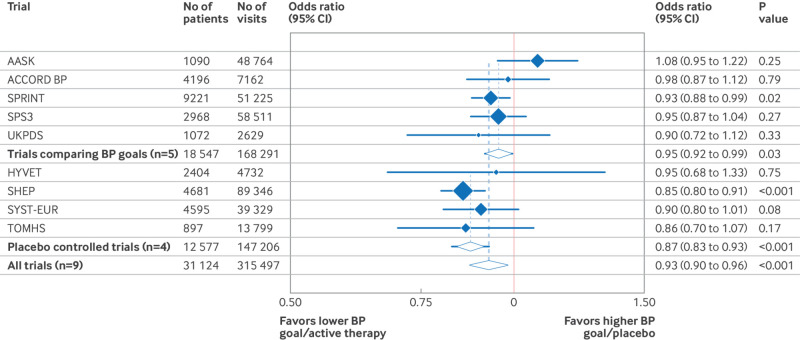
Effects of blood pressure (BP) treatment (either lower blood pressure treatment goal or active therapy versus higher blood pressure treatment goal or placebo) on occurrence of orthostatic hypertension at visit level, using generalized estimating equations to account for clustering by participant. Pooled effects are organized according to five blood pressure treatment goal trials and four placebo controlled trials and overall. Size of each point estimate and pooled effect is weighted by number of follow-up visits with orthostatic hypertension assessments. I^2^=38.0% (determined on basis of two stage meta-analysis, used to assess trial heterogeneity). CI=confidence interval

Although all trials, except AASK, showed a lower odds of orthostatic hypertension in either the lower goal or active arms, only SHEP and SPRINT had statistically significant results (fig 2). Pooling the five trials comparing blood pressure treatment goals showed that a lower (more intensive) treatment goal was associated with lower odds of orthostatic hypertension (odds ratio 0.95, 95% CI 0.92 to 0.99). Similarly, pooling together the placebo controlled trials showed that active therapy lowered the odds of orthostatic hypertension (odds ratio 0.87, 95% CI 0.83 to 0.93). We found moderate heterogeneity across the nine trials (I^2^=38.0%). When we pooled the nine trials together, a lower goal or active therapy reduced the odds of orthostatic hypertension compared with a higher goal or placebo (odds ratio 0.93, 95% CI 0.90 to 0.96). A sensitivity analysis using Poisson regression did not meaningfully change our findings (prevalence ratio 0.94, 95% CI 0.91 to 0.97). In sensitivity analyses using a two stage analysis, results were similar, both overall and by trial design (supplementary figures D-F).

We examined alternative definitions of orthostatic hypertension. With nearly all definitions examined, a lower (more intensive) blood pressure treatment goal or active therapy was associated with a lower odds ratio of orthostatic hypertension compared with a higher blood pressure treatment goal or placebo (table 3). We also examined the effect of treatment on orthostatic change as a continuous outcome variable (supplementary table G). Whereas trials of blood pressure treatment goal tended to increase orthostatic change in systolic blood pressure (that is, a trend toward an increase in systolic blood pressure with standing), placebo controlled trials tended to decrease orthostatic change in systolic or diastolic blood pressure. We also repeated the principal analyses using the recent consensus definition and the systolic change alone with similar results (see supplementary figures G-I). Finally, we examined alternate definitions of orthostatic hypertension with truncated centile ranges with similar results (supplementary table H).

**Table 3:** Effect of more intensive treatment on orthostatic hypertension, alternate definitions

Definition of orthostatic hypertension (mm Hg)	Lower BP goal or active therapy—No of visits (exposure/no exposure)	Higher BP goal or placebo—No of visits (exposure/no exposure)	Odds ratio (95% CI)	P value
**BP treatment goal trials (n=18 547)**				
ΔSBP ≥20 or ΔDBP ≥10 (primary definition)	19 833/73 827	19 982/72 472	0.95 (0.92 to 0.99)	0.03
ΔSBP ≥20	5612/88 048	5878/86 576	0.92 (0.86 to 0.99)	0.02
ΔDBP ≥10	17 837/75 823	17 748/74 706	0.97 (0.93 to 1.01)	0.11
ΔSBP ≥20 and standing SBP ≥140 (consensus definition)	4223/89 437	5370/87 084	0.71 (0.66 to 0.76)	<0.001
Standing SBP ≥140	26 559/67 101	45 988/46 466	0.31 (0.30 to 0.33)	<0.001
**Placebo controlled trials (n=12 577)**				
ΔSBP ≥20 or ΔDBP ≥10 (primary definition)	11 406/76 803	12 356/68 516	0.87 (0.83 to 0.93)	<0.001
ΔSBP ≥20	1503/86 706	1754/79 118	0.82 (0.73 to 0.92)	0.001
ΔDBP ≥10	10 673/77 536	11 501/69 371	0.88 (0.83 to 0.94)	<0.001
ΔSBP ≥20 and standing SBP ≥140 (consensus definition)	1375/86 834	1704/79 168	0.78 (0.69 to 0.88)	<0.001
Standing SBP ≥140	50 665/37 544	65 555/15 317	0.30 (0.28 to 0.32)	<0.001
**All trials (n=31 124)**				
ΔSBP ≥20 or ΔDBP ≥10 (primary definition)	31 239/150 630	32 338/140 988	0.93 (0.90 to 0.96)	<0.001
ΔSBP ≥20	7115/174 754	7632/165 694	0.90 (0.85 to 0.96)	<0.001
ΔDBP ≥10	28 510/153 359	29 249/144 077	0.94 (0.90 to 0.97)	<0.001
ΔSBP ≥20 and standing SBP ≥140 (consensus definition)	5598/176 271	7074/166 252	0.73 (0.68 to 0.77)	<0.001
Standing SBP ≥140	77 224/104 645	111 543/61 783	0.31 (0.30 to 0.32)	<0.001

Effects were determined via generalized estimating equations using binomial family logit link with robust variance estimator with adjustment for study.

Δ=change; BP=blood pressure; CI=confidence interval; DBP=diastolic blood pressure; SBP=systolic blood pressure.

### Stratified analyses

More intensive treatment (that is, a lower blood pressure treatment goal or active therapy) was associated with a lower odds of orthostatic hypertension than a higher blood pressure treatment goal or placebo in strata of age, sex, seated systolic blood pressure of ≥130 mm Hg or diastolic blood pressure of ≥80 mm Hg, estimated glomerular filtration rate <60 mL/min/1.73 m^2^, body mass index, history of cardiovascular disease, pre-randomization standing systolic blood pressure of ≥140 mm Hg, and pre-randomization orthostatic hypertension (table 4). We found evidence for greater reduction in the occurrence of orthostatic hypertension among non-black participants and participants without diabetes. Results were similar when we defined orthostatic hypertension by using the consensus definition (supplementary table I) and with truncated centiles (supplementary table J).

**Table 4:** Effect of more intensive treatment on orthostatic hypertension, stratified by pre-specified subgroups (all 9 trials)

Subgroups	No of participants	No of visits	Odds ratio (95% CI)	P value	P for interaction
Age:					
≤75 years	23 298	252 864	0.90 (0.86 to 0.93)	<0.001	0.71
>75 years	7822	62 559	0.88 (0.82 to 0.95)	0.001	
Sex:					
Male	16 365	168 933	0.92 (0.88 to 0.97)	0.001	0.06
Female	14 759	146 564	0.86 (0.82 to 0.91)	<0.001	
Race:					
Non-black	17 833	180 418	0.86 (0.83 to 0.90)	<0.001	0.003
Black	6292	91 018	0.97 (0.91 to 1.03)	0.35	
Pre-randomization SBP ≥130 or DBP ≥80 mm Hg*:					
No	3669	31 396	0.95 (0.86 to 1.04)	0.25	0.18
Yes	27 327	281 881	0.89 (0.85 to 0.92)	<0.001	
Diabetes:					
No	23 446	269 158	0.88 (0.85 to 0.91)	<0.001	0.05
Yes	7675	46 320	0.96 (0.89 to 1.04)	0.29	
Previous stroke:					
No	22 513	198 317	0.86 (0.83 to 0.90)	<0.001	0.05
Yes	3320	61 224	0.95 (0.87 to 1.03)	0.21	
Estimated eGFR:					
≥60 mL/min/1.73 m^2^	21 959	199 587	0.89 (0.86 to 0.93)	<0.001	0.77
<60 mL/min/1.73 m^2^	8583	106 563	0.90 (0.84 to 0.96)	0.002	
Body mass index:					
<30	19 849	205 648	0.89 (0.85 to 0.93)	<0.001	0.83
≥30	11 091	108 118	0.90 (0.85 to 0.95)	<0.001	
History of cardiovascular disease:					
No	25 548	237 618	0.88 (0.85 to 0.91)	<0.001	0.59
Yes	4473	28 849	0.90 (0.82 to 0.99)	0.03	
Standing SBP just before randomization*					
<140 mm Hg	26 842	285 204	0.89 (0.85 to 0.92)	<0.001	0.55
≥140 mm Hg	511	6153	0.96 (0.74 to 1.25)	0.75	
Pre-randomization orthostatic hypertension*:					
No	22 745	2377 05	0.89 (0.86 to 0.93)	<0.001	0.35
Yes	4569	52 916	0.93 (0.87 to 1.00)	0.04	

Effects were determined via generalized estimating equations using binomial family logit link with robust variance estimator in strata of baseline covariates with adjustment for study. Models were restricted to follow-up visits and included interaction terms between low goal assignment and stratum of interest.

CI=confidence interval; DBP=diastolic blood pressure; eGFR=estimated glomerular filtration rate; SBP=systolic blood pressure

* Based on visit in closest temporal proximity to randomization.

## Discussion

In this meta-analysis of individual participant data from 31 124 adults with elevated blood pressure and hypertension, more intensive treatment (that is, a lower blood pressure treatment goal or active therapy) modestly reduced the occurrence of orthostatic hypertension on the basis of measurements from 315 497 visits. This effect was consistent regardless of trial type or definition of orthostatic hypertension. Moreover, these effects were generally consistent across demographic characteristics and medical comorbidities.

### Comparison with other studies

Blood pressure is highly regulated by the autonomic nervous system in healthy adults such that blood pressure remains relatively constant across body positions. Whereas substantial focus has been directed toward falls in blood pressure on standing (that is, orthostatic hypotension), little attention has been given to orthostatic hypertension.^25^ ^26^ Recent epidemiological evidence has identified increases in blood pressure on standing as potentially pathologic, linking orthostatic hypertension with a range of adverse events, including cardiovascular disease, stroke, kidney disease, and cognitive impairment.^1^ ^2^ ^3^ These long term associations with adverse health outcomes contribute to a growing belief that orthostatic hypertension may represent a form of unrecognized or masked hypertension that may require monitoring of adults in the standing position and adjusting drug treatment accordingly.^27^

This study confirms that orthostatic hypertension is common among adults with hypertension, but it also shows that more intensive blood pressure treatment might attenuate orthostatic hypertension over time. This is an important observation. Our previous work showed that more intensive treatment caused a net increase in the difference in blood pressure in response to standing.^5^ However, this study suggests that this effect may be short term and dissipate with chronic treatment. This observation, if replicated, may be important for treating clinicians, who might be dissuaded from treating hypertension because of short term orthostatic hypertension. Physiologic mechanisms for this observation are unclear. The short term increase may be secondary to autonomic over-response to valsalva, cardioacceleration after leg muscle contraction, or mobilization of excess lower extremity fluid.^28^ ^29^ In the long term, we speculate that the resolution of orthostatic hypertension may be related to healthy remodeling of the vasculature with tighter blood pressure control.^30^ Further study of mechanisms should be evaluated in future work, particularly with repeat standing measurements. This may also be related to measurement error and additionally to more controlled blood pressure in general, such that blood pressure and fluctuation in blood pressure measurement is also lower,^31^ ^32^ reducing risk for orthostatic hypertension.

Our study used a definition of orthostatic hypertension that mirrored the one for orthostatic hypotension, used in previous work.^13^ ^14^ ^15^ ^16^ ^17^ However, discussion is ongoing as to how orthostatic hypertension should be defined.^25^ Unlike with orthostatic hypotension, which focuses on changes in blood pressure alone, recent guidelines have advocated for a definition that includes both an increase in systolic blood pressure on standing and an elevated standing threshold (systolic blood pressure ≥140 mm Hg).^18^ This definition was proposed for a general population, not necessarily a hypertensive population. When we used this definition, the prevalence of orthostatic hypertension was substantially lower in our population. Nevertheless, the effects of treatment were even more pronounced. This is due in part to our observation that antihypertensive agents, particularly the longer acting agents used in many of the trials in our study, lower blood pressure in all body positions.^5^ ^33^ As a result, standing hypertension would also be reduced with more intensive treatment. From the perspective of studying mechanisms of injury related to orthostatic hypertension, we caution against the use of this joint definition as it may make identifying and evaluating treatment response to the rise in blood pressure, which itself may be pathologic and occur below the 140 mm Hg threshold, more difficult.^17^ Some authors have also questioned whether diastolic blood pressure should be included in definitions of orthostatic hypertension, as diastolic blood pressure usually increases with standing.^34^ However, given that systolic and diastolic blood pressure are known to be correlated in both seated and standing positions, their change would be expected to correlate as well, and thus some patients with a rise in diastolic blood pressure would also have a rise in systolic blood pressure. Whether thresholds of change in systolic blood pressure or diastolic blood pressure are optimal for identifying risk with cardiovascular disease should be the focus of subsequent work. Nevertheless, the effects of treatment on orthostatic increases in systolic and diastolic blood pressure as defined in this study, using thresholds that mirrored those for orthostatic hypotension, were quite consistent.

We did not identify compelling evidence that the effects of treatment differed by demographic or medical characteristics. Although a strong interaction between black and non-black populations was apparent, this information was not uniformly collected by trials outside of the US, reducing our sample for this analysis. We also observed that effects were attenuated among adults with diabetes. Mechanisms are beyond the scope of this study, but we speculate that this lack of effects among black adults and those with diabetes may reflect known challenges in achieving blood pressure control. Additional research should probe these associations further.

### Limitations and strengths of study

Our study has limitations. Firstly, we identified only nine trials, which differed with respect to their interventions, frequency of follow-up, duration, blood pressure measurement procedures, and study populations. These differences might have influenced our results. Despite these differences, our findings were relatively consistent and our sample was sufficiently large that additional trials are not likely to alter our pooled observation. Secondly, generalizability to clinical practice may be limited owing to the strict entry criteria used by these trials, differences in prescribing regimens that might not reflect real world drug choices, and careful monitoring and drug titration protocols that may affect titration patterns. Thirdly, subgroup analyses relied on covariate definitions that were based on self-report and at times differed in definition across studies. Any resulting misclassification could weaken contrasts across subgroups, reducing our ability to detect differences. Moreover, we pre-specified our subgroups. Whether associations might differ across different categories (for example, younger age) should be examined in dedicated studies. Fourthly, we did not examine antihypertensive drug class in this study, which should be a focus of subsequent work. Fifthly, assessments of orthostatic hypertension were based on seated-to-standing protocols, which may not be interchangeable with supine-to-standing maneuvers.^29^ ^33^ ^35^ In the case of orthostatic hypotension, some authors have proposed modified thresholds for identifying adults with orthostatic hypotension (that is, a drop in systolic blood pressure of 15 mm Hg or diastolic blood pressure of 7 mm Hg).^36^ In our own work, we have observed a net increase in blood pressure with standing from the seated position.^29^ Thus, whether a seated-to-standing protocol should have a higher or lower threshold compared with supine-to-standing to establish orthostatic hypertension remains unclear. Moreover, aside from SYST-EUR, none of the major outcome trials examined in our study measured supine blood pressure.^33^ The manner by which starting position might underestimate or overestimate orthostatic hypertension should be the focus of future work. Sixthly, although both lower treatment goals and active therapy lowered the occurrence of orthostatic hypertension, the effect of active therapy was greater in magnitude. Whether different goals might alter the observed effect is beyond the scope of this study. Seventhly, temporal effects of treatment on orthostatic hypertension should be interpreted cautiously, as different populations contributed to visits at different time points. We attempted to include all available data to preserve the trials’ randomized contrasts. However, as some trials (for example, ACCORD) assessed orthostatic blood pressure after starting, some of these participants did not have a pre-randomization assessment, which could influence estimates of orthostatic hypertension at baseline. This would not affect the pooled contrast overall but could affect the proportion with orthostatic hypertension over time. Finally, our analysis did not examine the effects of treatment on clinical events among adults with orthostatic hypertension. This has been questioned in previous work and represents an important focus for subsequent research.^14^

This study has notable strengths. Firstly, this systematic review and meta-analysis represents one of the largest data collections of orthostatic hypertension in the context of drug treatment for hypertension. Secondly, doing an individual participant meta-analysis allowed for greater harmonization of data and examination of under-represented subgroups that were not feasible within individual trials. Thirdly, whereas data on orthostatic hypertension have been presented from various trials with respect to outcomes, to our knowledge this is the only patient level meta-analysis of the risk of orthostatic hypertension in treated hypertensive patients. Finally, the associations between treatment and orthostatic hypertension across trials were similar, suggesting that the effects of more intensive hypertension treatment on orthostatic hypertension are quite reproducible.

### Implications

Our study potentially has clinical implications. Orthostatic hypertension has received increasing attention as a novel and distinct presentation of hypertension. This carries the suggestion that distinct pharmacologic strategies are needed for its treatment. Some authors have suggested that β blockers may be more effective for treating orthostatic hypertension by blunting the β adrenergic response to standing, and evidence also shows that peripheral α blockers may be effective.^6^ However, neither of these classes is preferred for initial treatment of hypertension on the basis of the experience from hypertension outcome trials.^37^ Nevertheless, if either is superior to other classes in reducing orthostatic hypertension, it might be of value as add-on therapy in the presence of residual orthostatic hypertension. Although more work is needed to evaluate specific drug classes with respect to orthostatic hypertension, our data provide reassurance that focusing on seated blood pressure control and treating seated hypertension among adults with hypertension can modestly reduce orthostatic hypertension. At this time, no trials are assessing whether treating standing blood pressure levels to some goal provides additional benefit to the traditional seated approach.

### Conclusions

In this large, individual participant data meta-analysis of blood pressure treatment trials, more intensive blood pressure treatment, especially with active treatment (versus placebo), modestly reduced the occurrence of orthostatic hypertension regardless of its definition or baseline demographic and medical characteristics. Future research should examine orthostatic hypertension in relation to clinical outcomes as well as whether specific classes of antihypertensive drugs or lower treatment goals might better prevent orthostatic hypertension and its sequelae.

## What is already known on this topic

Orthostatic hypertension (an extreme increase in blood pressure after standing) is a pathologic form of higher standing blood pressure, predicting adverse health outcomes in observational studies
Current recommendations to treat orthostatic hypertension are based on a few small trials of agents not considered first line for hypertension treatment

## What this study adds

Using data from nine randomized trials of >30 000 participants, this individual level meta-analysis shows that more intensive blood pressure treatment reduces the occurrence of orthostatic hypertension
Many of the included trials used first line antihypertensive agents, suggesting that common approaches for seated hypertension may also be used to treat orthostatic hypertension
Although orthostatic hypertension is common among adults with hypertension, it may be treated using standard approaches recommended for seated hypertension

## Data Availability

The data associated with this paper were used with institutional agreements between the NHLBI BioLINCC repository of institutions that conducted the original trials. The data use agreements do not permit public sharing of trial data. However, most of the data used in these analyses may be obtained with data use agreements involving the NHLBI BioLINCC or by request to the corresponding authors of the original trials. A dummy dataset and corresponding analytic codes are available at https://doi.org/10.7910/DVN/RHUF9F.

## Web extra

Extra material supplied by authors

Web appendix: Supplementary methods

Web appendix: Supplementary figures and tables

## Notes

Contributors: SPJ contributed to oversight, conceptualization, resources, data extraction, data analysis, data interpretation, primary draft, and critical review of the manuscript. J-RH contributed to data extraction and critical review of the manuscript. JLC contributed to data extraction and critical review of the manuscript. CM contributed to search strategy, support, and critical review of the manuscript. LAL contributed to data interpretation and critical review of the manuscript. LJA, NSB, BRD, RRH, ERM, RP, JAS, AAT, and JTW contributed to primary data acquisition, data interpretation, and critical review of the manuscript. KJM contributed to conceptualization, data interpretation, and critical review of the manuscript. WCC contributed to project oversight, conceptualization, data interpretation, primary draft, and critical review of the manuscript. SPJ had full access to all the data in the study and takes responsibility for the integrity of the data and the accuracy of the data analysis. SPJ is the guarantor. The corresponding author attests that all listed authors meet authorship criteria and that no others meeting the criteria have been omitted.

Transparency: The lead author (the manuscript’s guarantor) affirms that the manuscript is an honest, accurate, and transparent account of the study being reported; that no important aspects of the study have been omitted; and that any discrepancies from the study as planned (and, if relevant, registered) have been explained.

Dissemination to participants and related patient and public communities: An abstract of this research was presented at the AHA Hypertension Conference in September 2023 and will be publicly available through PubMed Central for patients and clinicians throughout the world.

Provenance and peer review: Not commissioned; externally peer reviewed.
