# Supplementary material for: Effects of intensive blood pressure treatment on orthostatic hypertension: individual level meta-analysis
Source: BMJ. 2025 Mar 25;388:e080507. doi: 10.1136/bmj-2024-080507 (PMC11934097; doi:10.1136/bmj-2024-080507)
Supplement: Supplementary file 1 — Web appendix: Supplementary methods [file jurs080507.ww1.pdf]

Supplementary Figures and Tables

Supplementary Figure A. PRISMA Diagram

Supplementary Figure B. Distributions of systolic blood pressure in seated and standing positions and the difference between positions

Supplementary Figure C. Distributions of diastolic blood pressure in seated and standing positions and the difference between positions

Supplementary Figure D. Two-stage meta-analysis, effects of more intensive treatment on orthostatic hypertension (all nine trials)

Supplementary Figure E. Two-stage meta-analysis, effects of more intensive treatment on orthostatic hypertension (trials comparing blood pressure treatment goals)

Supplementary Figure F. Two-stage meta-analysis, effects of more intensive treatment on orthostatic hypertension (trials comparing active therapy with placebo)

Supplementary Figure G. Proportion of participants with orthostatic hypertension (OHTN) by study month, using the consensus definition.

Supplementary Figure H. Effects of blood pressure treatment on the occurrence of orthostatic hypertension, defined using the consensus definition.

Supplementary Figure I. Effects of blood pressure treatment on the occurrence of orthostatic hypertension, defined using the systolic change alone (regardless of change in diastolic blood pressure).

Supplementary Table A. Risk of bias according to trial

Supplementary Table B. Values of extreme percentiles, truncated at 0.01^{st} and 99.99^{th} percentiles

Supplementary Table C. Values of extreme percentiles, truncated at 0.1^{st} and 99.9^{th} percentiles

Supplementary Table D. Comparison of participants with and without orthostatic hypertension assessments at baseline

Supplementary Table E. Mean blood pressure before and after randomization by assignment

Supplementary Table F. Effects of more intensive blood pressure treatment on orthostatic hypertension by follow-up time

Supplementary Table G. Effects of treatment on orthostatic change in systolic or diastolic blood pressure

Supplementary Table H. Effect of treatment on alternate definitions of orthostatic hypertension, using truncated percentiles

Supplementary Table I. Subgroup analyses using the orthostatic hypertension consensus definition

Supplementary Table J. Subgroup analyses using the primary orthostatic hypertension definition with truncated percentiles

Supplementary Figure A. PRISMA diagram depicting most recent search strategy in the context of prior search results.

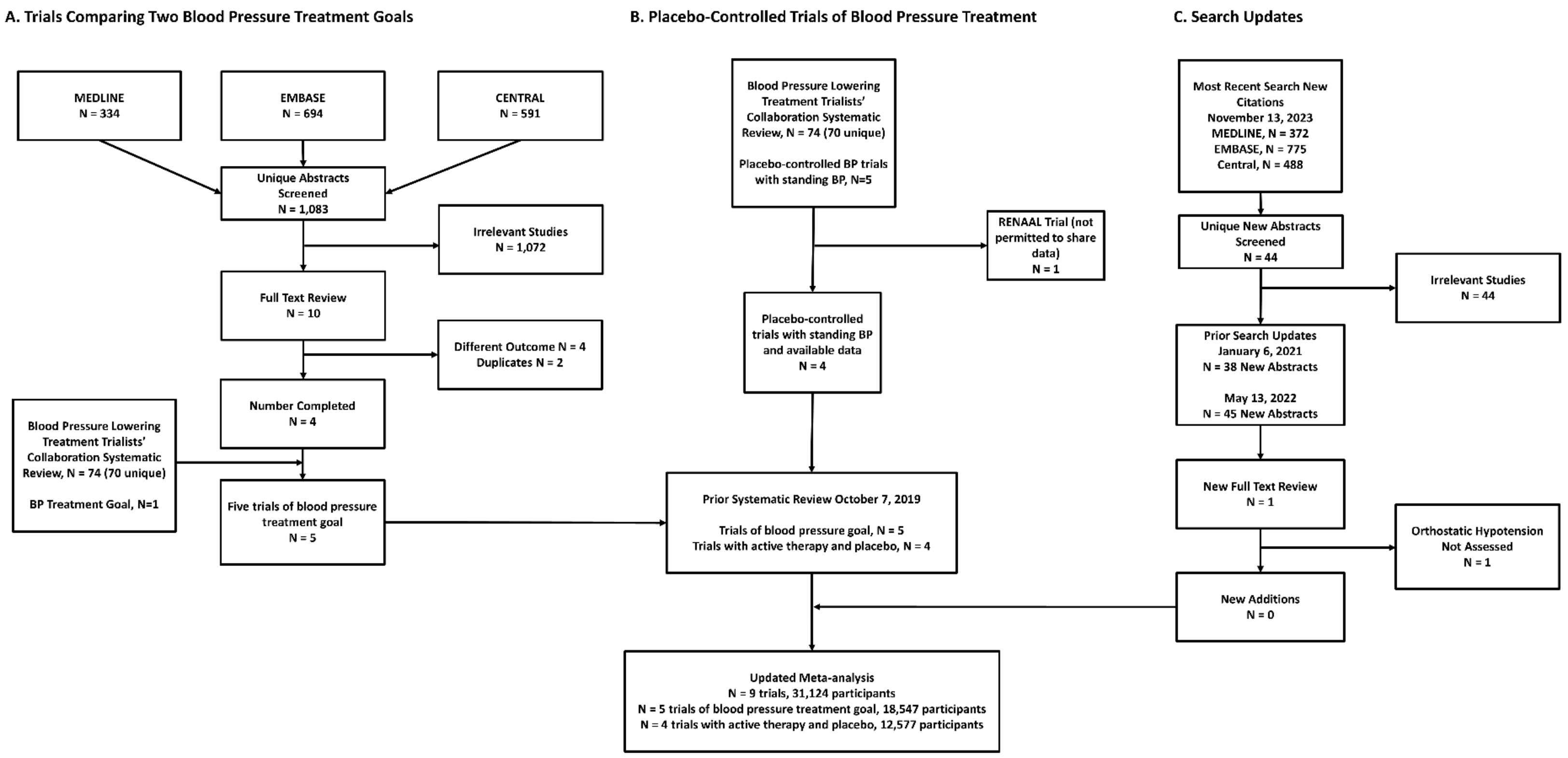

Supplementary Figure B. Kernel density plots (bandwidth 5) of the distribution (y-axis) of (**A**) seated systolic blood pressure (SBP), (**B**) standing SBP, and (**C**) postural change in SBP. Curves represent blood pressure measurements (mm Hg) during baseline (solid line), standard blood pressure treatment goal or placebo follow-up visits (dashed line), and lower blood pressure treatment goal or active therapy follow-up visits (dotted line) for all 9 trials. These figures are restricted to the subpopulation with both baseline and follow-up visits (N=27,314). Note that some trials enrolled participants with treated hypertension, which may account for normal seated blood pressure values at baseline.

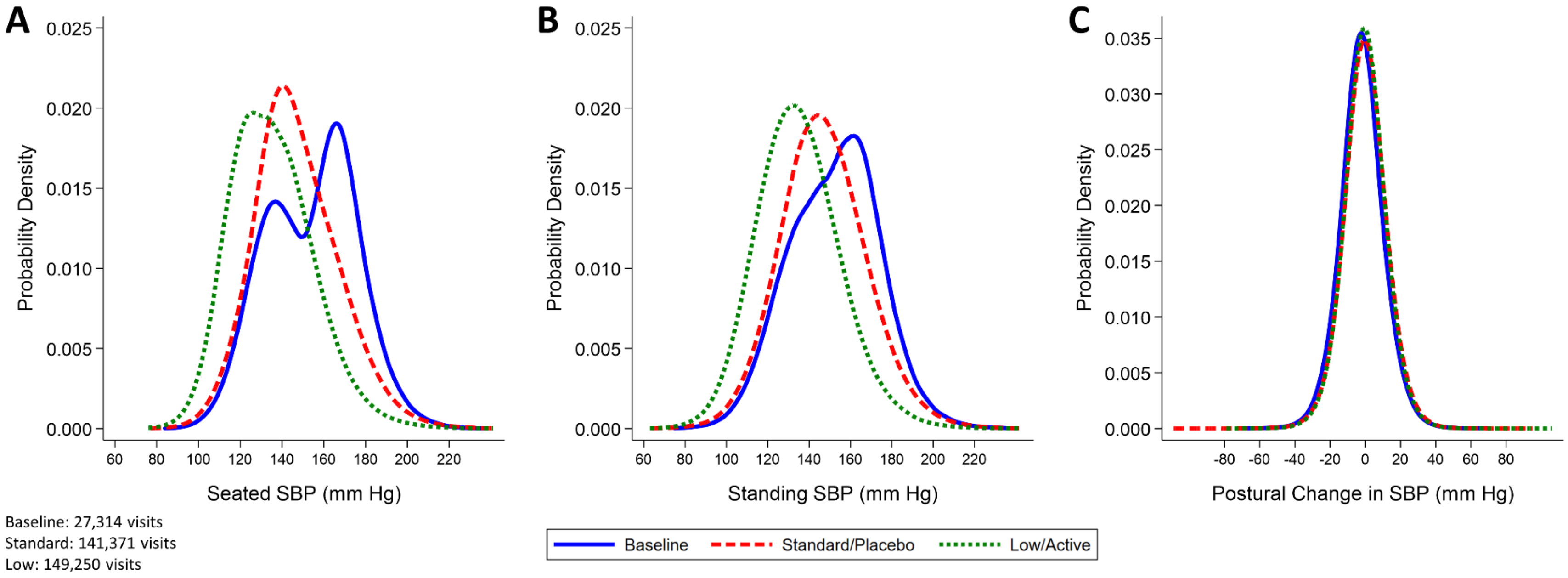

Supplementary Figure C. Kernel density plots (bandwidth 5) of the distribution (y-axis) of (**A**) seated diastolic blood pressure (DBP), (**B**) standing DBP, and (**C**) postural change in DBP. Curves represent blood pressure measurements (mm Hg) during baseline (solid line), standard blood pressure treatment goal or placebo follow-up visits (dashed line), and low blood pressure treatment goal or active therapy follow-up visits (dotted line) for all 9 trials. These figures are restricted to the subpopulation with both baseline and follow-up visits (N=27,314). Note that some trials enrolled participants with treated hypertension, which may account for normal seated blood pressure values at baseline.

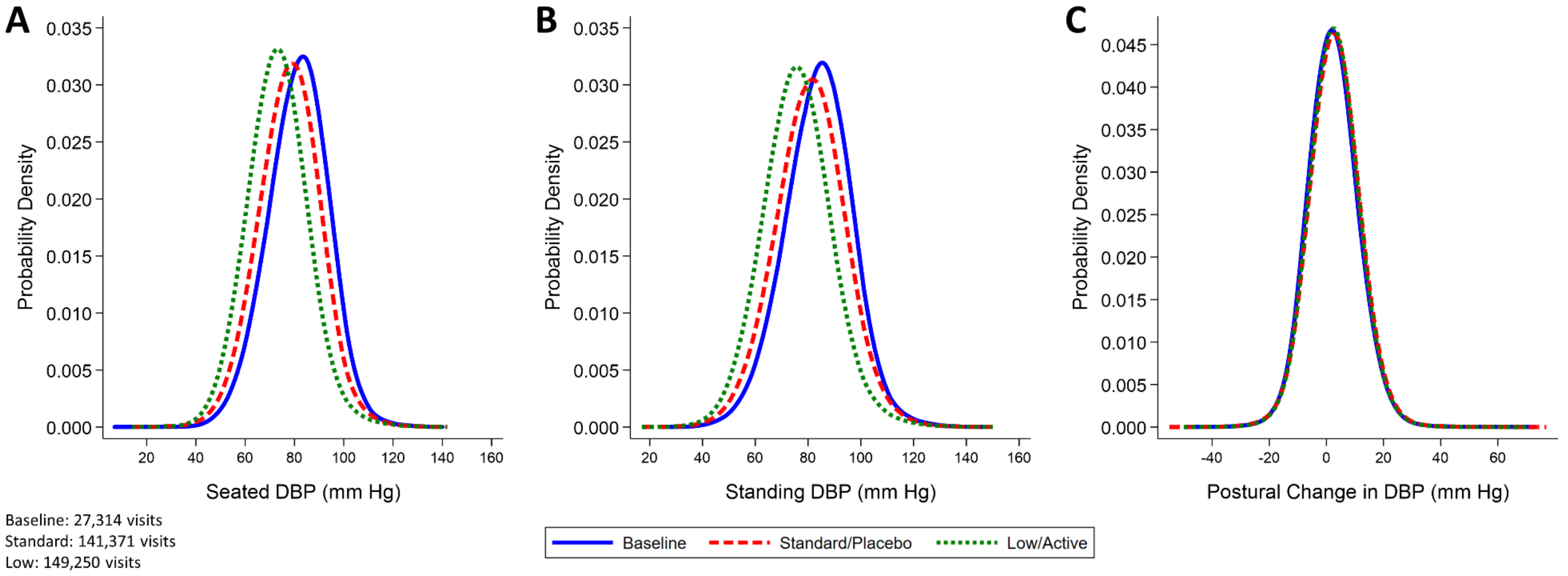

Supplementary Figure D. Two-stage meta-analysis of the effects of more intensive treatment on orthostatic hypertension. Individual effects were determined based on generalized estimating equations (binomial family, logit link, robust variance estimator). The pooled effect from the 9 trials were weighted with the inverse variance, using a random effects model.

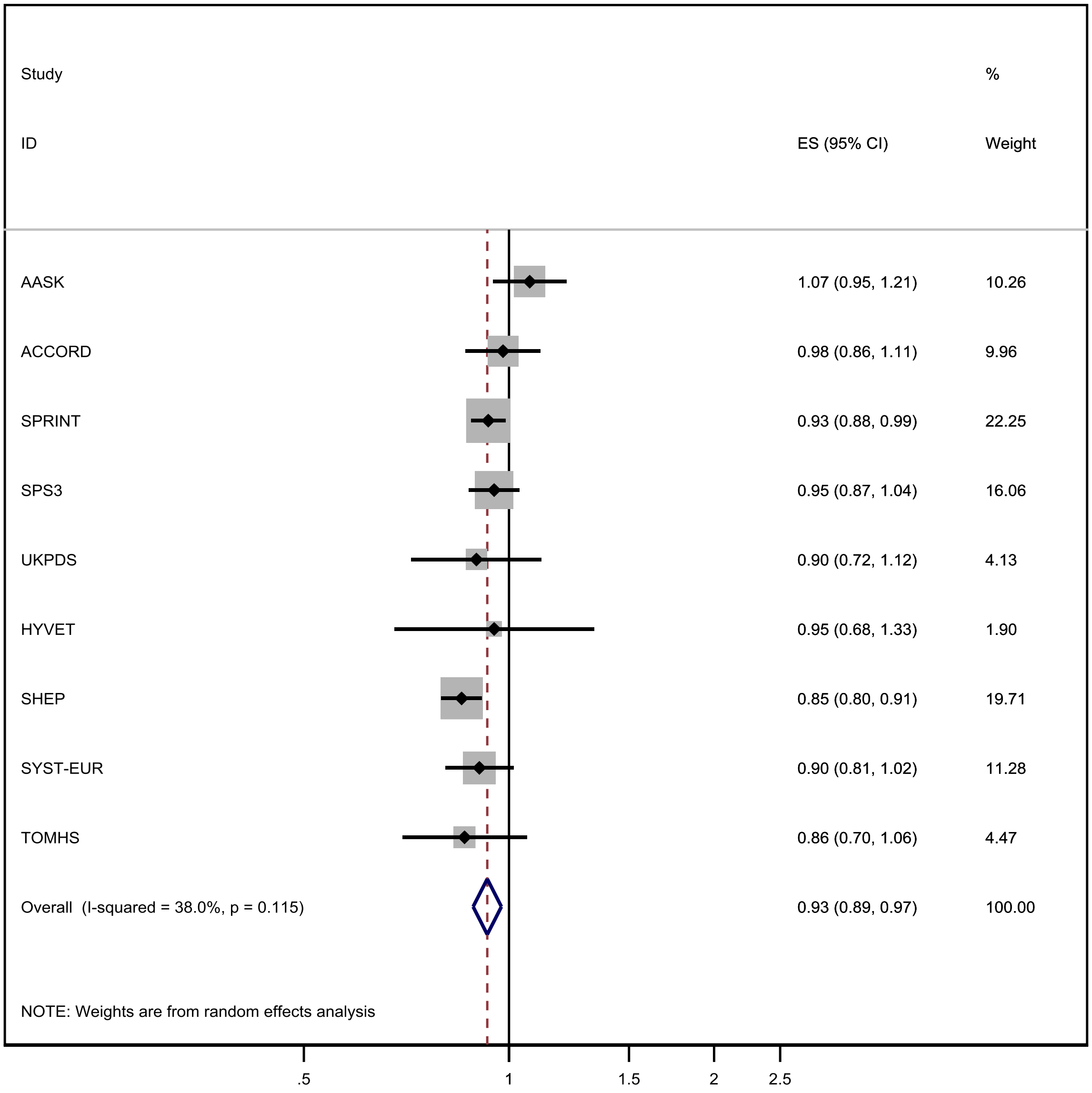

Supplementary Figure E. Two-stage meta-analysis of the effects of more intensive treatment on orthostatic hypertension. Individual effects were determined based on generalized estimating equations (binomial family, logit link, robust variance estimator). The pooled effect from the 5 trials comparing blood pressure treatment goals were weighted with the inverse variance, using a random effects model.

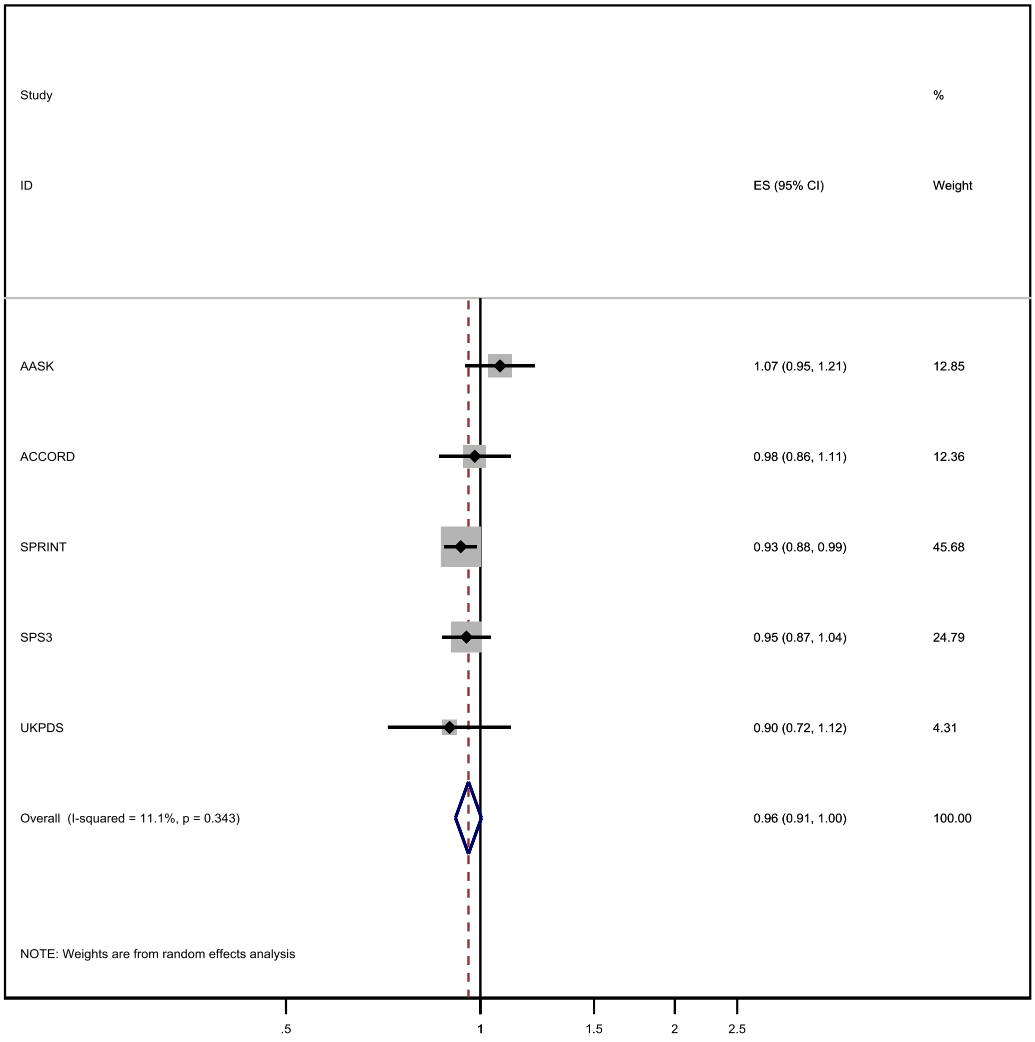

Supplementary Figure F. Two-stage meta-analysis of the effects of more intensive treatment on orthostatic hypertension. Individual effects were determined based on generalized estimating equations (binomial family, logit link, robust variance estimator). The pooled effect from the 4 trials comparing active therapy with placebo were weighted with the inverse variance, using a random effects model.

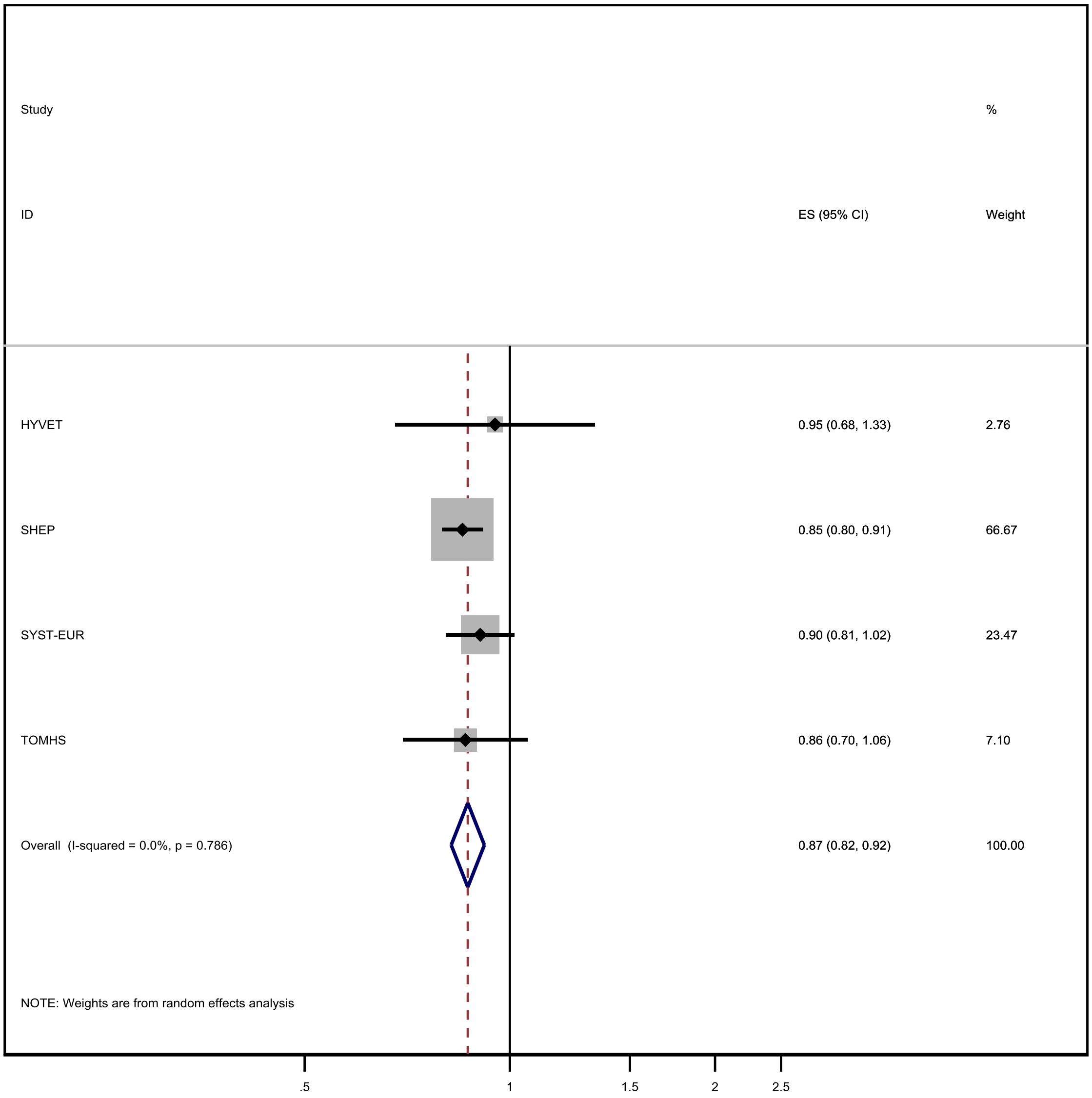

Supplementary Figure G. Proportion of participants with orthostatic hypertension (OHTN) based on the consensus definition by study month. The consensus definition for orthostatic hypertension combines an orthostatic change in systolic blood pressure of at least 20 mm Hg with a standing systolic blood pressure of ≥140 mm Hg. Follow-up visits were grouped together (month 0/prior randomization, <1 month, 1-6 months, 6-12 months, 12-24 months, 24-36 months, 36-48 months, and greater than 48 months). The lines represent the aggregate, pooled proportion across the 9 trials (circle, a higher blood pressure (BP) treatment goal/placebo; diamond, a lower BP treatment goal/active therapy). The vertical capped thick lines represent 95% confidence intervals (CI). Proportions were estimated via generalized estimating equations, using an unadjusted Poisson family, log link. The numbers in the table represent the measurements contributing to each proportion by time period. Note that some trials had multiple visits prior to randomization, which contributed to these models and may account for differences in the proportion of OHTN estimated via this approach, versus the descriptive estimate based on a single visit in Table 2. In addition, we report the number of unique participants at risk in each time period and the % with OHTN at any time during this time period. These proportions differ from the figure.

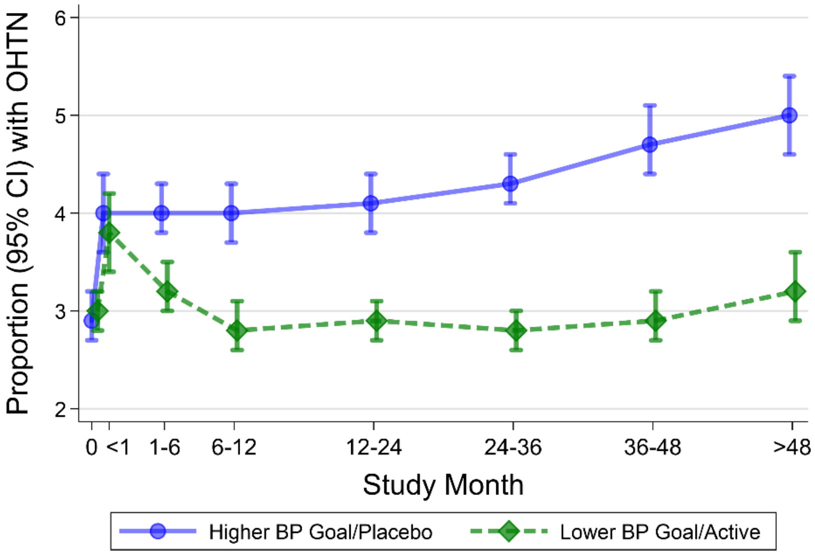

**N of Measures**	**0**	**<1**	**1-6**	**6-12**	**12-24**	**24-36**	**36-48**	**>48**
**Higher BP Goal/Placebo**	19,312	10,244	25,322	23,659	30,351	24,441	20,613	19,384
**Lower BP Goal/Active**	20,386	10,617	25,132	22,820	32,227	26,386	23,174	21,127

**N of Participants at Risk (% OHTN at any time)**								
**Higher BP Goal/Placebo**	13,265 (3.6)	8,775 (4.8)	10,197 (8.2)	12,058 (6.6)	9,232 (9.8)	7,743 (10.2)	8,082 (9.4)	6,048 (11.1)
**Lower BP Goal/Active**	14,154 (3.7)	9,174 (4.4)	10,652 (6.4)	12,709 (4.3)	10,104 (7.2)	8,465 (6.9)	9,098 (6.3)	6,622 (8.1)

Supplementary Figure H Effects of blood pressure treatment (either a lower blood pressure treatment goal or active therapy versus a higher blood pressure treatment goal or placebo) on the occurrence of orthostatic hypertension, defined using the consensus definition, at the visit level, using generalized estimating equations to account for clustering by participant. The consensus definition for orthostatic hypertension combines an orthostatic change in systolic blood pressure of at least 20 mm Hg with a standing systolic blood pressure of ≥140 mm Hg. Circles represent odds ratios and horizontal lines represent 95% confidence intervals by study. Pooled effects (diamonds) are organized according to the 5 blood pressure treatment goal trials and the 4 placebo-controlled trials and overall. The size of each point estimate and pooled effect is weighted by the number of follow-up visits with orthostatic hypertension assessments. The I^2 statistic was determined based on a two-stage meta-analysis, used to assess trial heterogeneity.

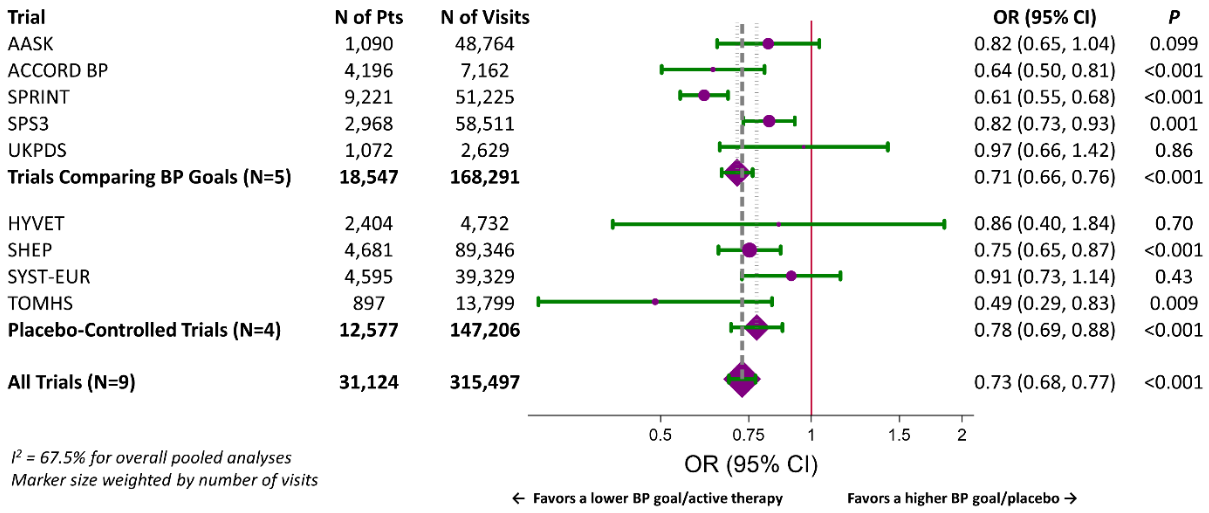

Supplementary Figure I. Effects of blood pressure treatment (either a lower blood pressure treatment goal or active therapy versus a higher blood pressure treatment goal or placebo) on the occurrence of orthostatic hypertension, defined using the orthostatic change in systolic blood pressure of at least 20 mm Hg alone (regardless of change in diastolic blood pressure) at the visit level, using generalized estimating equations to account for clustering by participant. Circles represent odds ratios and horizontal lines represent 95% confidence intervals by study. Pooled effects (diamonds) are organized according to the 5 blood pressure treatment goal trials and the 4 placebo-controlled trials and overall. The size of each point estimate and pooled effect is weighted by the number of follow-up visits with orthostatic hypertension assessments. The I^2 statistic was determined based on a two-stage meta-analysis, used to assess trial heterogeneity.

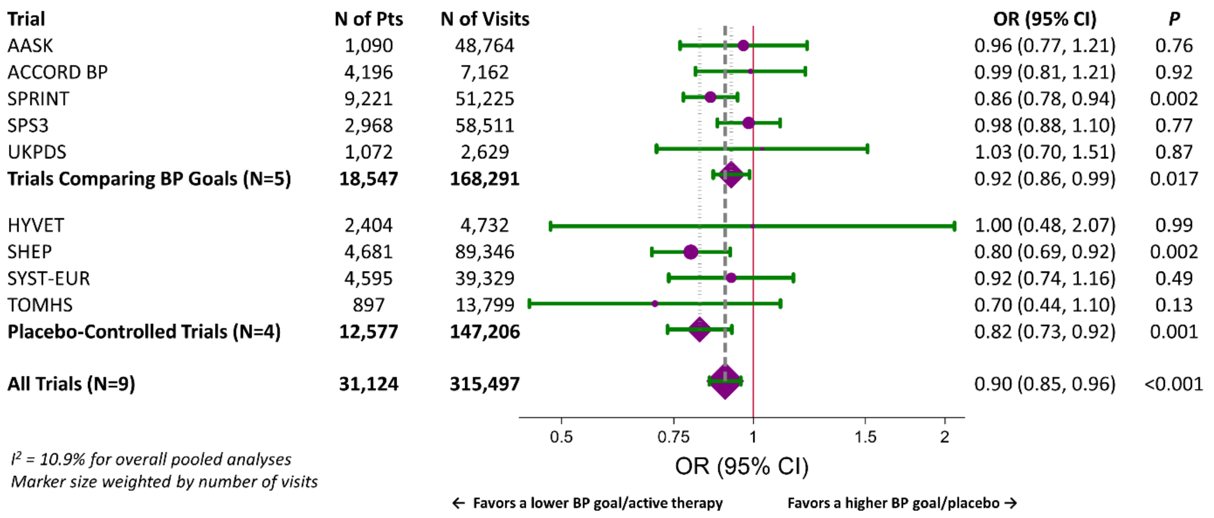

Supplementary Table A. Risk of Bias Table

Trial Name	Random sequence generation (selection bias)	Allocation concealment (selection bias)	Blinding of participants and personnel* (performance bias)	Blinding of outcome assessment* (detection bias)	Incomplete outcome data** (attrition bias)	Selective reporting (reporting bias)
**Trials of Blood Pressure Goal**						
AASK	Low	Low	Unclear	Unclear	Low	Low
ACCORD BP	Low	Low	Unclear	Unclear	Low	Low
SPRINT	Low	Low	Unclear	Unclear	Low	Low
SPS3	Low	Low	Unclear	Unclear	Unclear	Low
UKPDS	Low	Low	Unclear	Unclear	Low	Low
**Trials of Active Therapy versus Placebo**						
HYVET	Low	Low	Low	Low	Low	Low
SHEP	Low	Low	Low	Low	Unclear/Low	Low
SYST-EUR	Low	Low	Low	Low	Unclear	Low
TOMHS	Low	Low	Low	Low	Low	Low

*The primary outcome of this meta-analysis was orthostatic hypertension, which was not a focus these trials. Thus, while the outcome was not blinded for participants or data assessors, it was not an outcome they were monitoring.

**This was based on the degree of missing outcome data and reporting of attrition by treatment assignment (versus for the whole study).

Updated from *Juraschek et al, Annals 2021* to reflect the outcome of the present study, orthostatic hypertension.

Supplementary Table B. Values corresponding to extreme percentiles in the combined dataset

	Percentile	
**Factor**	**0.01**	**99.99**
Baseline body mass index, kg/m^2	13.70	78.36
Baseline estimated glomerular filtration rate, mL/min per 1.73 m^2	12.63	139.06
Baseline seated systolic blood pressure, mm Hg	80.00	238.00
Baseline seated diastolic blood pressure, mm Hg	6.00	141.00
Baseline standing systolic blood pressure, mm Hg	73.47	242.00
Baseline standing diastolic blood pressure, mm Hg	18.00	150.00
Seated systolic blood pressure (baseline and follow-up), mm Hg	75.00	242.00
Seated diastolic blood pressure (baseline and follow-up), mm Hg	13.00	143.00
Standing systolic blood pressure (baseline and follow-up), mm Hg	62.00	245.00
Standing diastolic blood pressure (baseline and follow-up), mm Hg	16.80	152.00

Supplementary Table C. Values corresponding to less extreme percentiles in the combined dataset

	Percentile	
**Factor**	**0.1**	**99.9**
Baseline estimated glomerular filtration rate, mL/min per 1.73 m^2	16.52	128.96
Baseline body mass index, kg/m^2	16.41	54.80
Baseline seated systolic blood pressure, mm Hg	95.30	218.00
Baseline seated diastolic blood pressure, mm Hg	35.00	129.00
Baseline standing systolic blood pressure, mm Hg	86.00	224.00
Baseline standing diastolic blood pressure, mm Hg	40.00	138.00
Seated systolic blood pressure (baseline and follow-up), mm Hg	86.00	219.00
Seated diastolic blood pressure (baseline and follow-up), mm Hg	36.00	123.00
Standing systolic blood pressure (baseline and follow-up), mm Hg	80.00	221.00
Standing diastolic blood pressure (baseline and follow-up), mm Hg	37.03	130.00

Supplementary Table D. Participant characteristics with and those missing pre-randomization orthostatic hypertension

	With pre-randomization orthostatic hypertension		Without pre-randomization orthostatic hypertension	
**Characteristic**	**N**	**Mean (SD) or %**	**N**	**Mean (SD) or %**
Age, years	27310	68.3 (10.6)	3810	63.1 (7.8)
Age > 75 years, %	27310	27.5	3810	8.1
Women, %	27314	47.7	3810	45.5
Black, %	20318	26.6	3807	23.4
Pre-randomization seated SBP, mm Hg	27314	154.2 (21.4)	3674	141.1 (16.7)
Pre-randomization seated DBP, mm Hg	27314	81.5 (11.5)	3656	76.6 (10.7)
eGFR*, mL/min per 1.73 m^2	21340	70.3 (19.8)	3599	82.9 (18.5)
Stage III CKD*, %	26808	30.2	3734	13.3
Body mass index, kg/m^2	27149	28.6 (5.5)	3791	31.4 (5.7)
Obese, %	27149	33.1	3791	55.8
Diabetes, %	27312	16.3	3809	84.4
Prior stroke, %	25010	10.7	823	79.5
History of CVD, %	26219	12.9	3802	28.6

Note: the majority of participants without baseline orthostatic hypertension assessments were from the ACCORD trial due to late implementation of their standing blood pressure protocol. While these participants may not have contributed to the pre-randomization assessment, they did contribute to follow-up assessments

Abbreviations: CKD, chronic kidney disease; DBP, diastolic blood pressure; eGFR, estimated glomerular filtration rate; SBP, systolic blood pressure; SD, standard deviation

Supplementary Table E. Means (SE) blood pressure measurements before and after randomization

	Pre-randomization, N= 27,419 participants with 39,698 measurements		Post-randomization, N= 31,124 participants with 315,497 measurements	
	Seated	Standing	Seated	Standing
Systolic blood pressure				
Higher BP goal/Placebo	154.5 (0.18)	152.8 (0.18)	145.5 (0.13)	145.8 (0.13)
Lower BP goal/Active Therapy	154.2 (0.18)	152.4 (0.18)	133.8 (0.13)	134.0 (0.12)
Diastolic blood pressure				
Higher BP goal/Placebo	80.8 (0.10)	83.4 (0.10)	77.3 (0.08)	80.1 (0.08)
Lower BP goal/Active Therapy	81.2 (0.09)	83.5 (0.10)	72.5 (0.07)	75.1 (0.08)

Estimated via generalized estimating equations with a normal distribution, identity link, and robust variance estimator. Models were unadjusted.

There were 15,123 participants with 173,326 measurements assigned a lower BP goal/Active therapy and 16,001 participants with 181,869 measurements assigned a higher BP goal/Placebo.

Abbreviations: BP, blood pressure; SE, standard error

Supplementary Table F. The odds of orthostatic hypertension by follow-up time according to treatment assignment for all 9 trials (31,124 participants with 355,195 orthostatic hypertension assessments).

	Lower BP Goal/Active Therapy			Higher BP Goal/Placebo			Interaction	
**Follow-up time**	**Proportion with OHTN**	**Difference in Proportion**	**OR (95% CI)**	**Proportion with OHTN**	**Difference in Proportion**	**OR (95% CI)**	**OR (95% CI)**	***P*-interaction**
Pre-randomization	16.9	*Ref*	*Ref*	15.5	*Ref*	*Ref*	*Ref*	
≤1 month	18.9	2.0 (1.1 to 2.9)	1.16 (1.10 to 1.23)	19.1	3.7 (2.8 to 4.5)	1.02 (0.96 to 1.08)	1.14 (1.05 to 1.24)	0.001
>1 to ≤6 months	19.1	2.3 (1.6 to 2.9)	1.14 (1.08 to 1.19)	18.0	2.6 (1.9 to 3.2)	1.09 (1.04 to 1.14)	1.04 (0.98 to 1.12)	0.22
>6 to ≤12 months	18.1	1.3 (0.6 to 1.9)	1.03 (0.98 to 1.09)	16.2	0.8 (0.1 to 1.4)	1.05 (1.00 to 1.11)	0.98 (0.91 to 1.05)	0.61
>12 to ≤24 months	17.9	1.0 (0.3 to 1.7)	1.01 (0.96 to 1.06)	16.1	0.6 (-0.0 to 1.3)	1.02 (0.97 to 1.07)	0.99 (0.93 to 1.06)	0.82
>24 to ≤36 months	18.5	1.6 (0.8 to 2.3)	0.99 (0.94 to 1.05)	16.0	0.5 (-0.2 to 1.2)	1.04 (0.99 to 1.10)	0.95 (0.88 to 1.03)	0.20
>36 to ≤48 months	18.8	1.9 (1.1 to 2.7)	1.05 (0.99 to 1.11)	16.9	1.5 (0.7 to 2.2)	1.05 (0.99 to 1.11)	1.00 (0.93 to 1.08)	0.97
>48 months	18.9	2.0 (1.1 to 2.9)	1.05 (0.99 to 1.12)	17.2	1.8 (0.9 to 2.6)	1.04 (0.98 to 1.11)	1.01 (0.93 to 1.10)	0.86

Abbreviations: BP, blood pressure; CI, confidence interval; OHTN, orthostatic hypertension; OR, odds ratio

Note: There 15,123 participants assigned to a standard blood pressure treatment goal with 173,326 visits (including baseline), and 16,001 participants assigned to an intensive blood pressure treatment goal with 181,869 visits (including baseline). Effects were determined via generalized estimating equations using a binomial family logit link with a robust variance estimator with a visit-term in strata of assignment. Proportions and difference in proportions were unadjusted, while ORs were adjusted for study. An interaction term was used to a compare effects across visit times. In this table, an OR>1 indicates that orthostatic hypertension was more frequent than the pre-randomization period. Pre-randomization estimates were model-generated and are based on all available pre-randomization visits. As a result the estimates here differ slightly from the estimates presented in Table 2.

Supplementary Table G. Effects of treatment on orthostatic change in systolic or diastolic blood pressure

	N of Measurements	β (95% CI), mm Hg	SE	*P*
**BP Treatment Goal Trials, N=18,547**				
ΔSBP, mm Hg	168291	0.24 (0.01 to 0.47)	0.12	0.04
ΔDBP, mm Hg	168291	-0.01 (-0.15 to 0.13)	0.07	0.91
**Placebo-Controlled Trials, N=12,577**				
ΔSBP, mm Hg	147206	-0.22 (-0.43 to -0.01)	0.11	0.04
ΔDBP, mm Hg	147206	-0.22 (-0.36 to -0.08)	0.07	0.002
**All Trials, N=31,124**				
ΔSBP, mm Hg	315497	0.04 (-0.12 to 0.21)	0.08	0.59
ΔDBP, mm Hg	315497	-0.09 (-0.20 to 0.01)	0.05	0.07

Effects were determined via generalized estimating equations using a normal family identity link with a robust variance estimator adjusted for study. The beta coefficient is the mean orthostatic change in blood pressure. Abbreviations: BP, blood pressure; CI, confidence intervals; DBP, diastolic blood pressure; SE, standard error; SBP, systolic blood pressure

Supplementary Table H. Effect of treatment on alternate definitions of orthostatic hypertension, using truncated percentiles to exclude extreme blood pressure values (0.1st and 99.9th)

Definition of OHTN (mm Hg)	Lower BP Goal or Active Therapy, N of Visits (Exposure/No Exposure)	Higher BP Goal or Placebo, N of Visits (Exposure/No Exposure)	OR (95% CI)	*P*
**BP Treatment Goal Trials, N=18,547**				
ΔSBP≥20 or ΔDBP≥10	19661/73224	19864/72124	0.96 (0.92 to 1.00)	0.03
ΔSBP≥20	5565/87643	5856/86377	0.92 (0.86 to 0.98)	0.01
ΔDBP≥10	17750/75544	17672/74511	0.97 (0.93 to 1.01)	0.12
ΔSBP≥20 & Standing SBP≥140 (recent consensus definition)	4201/89007	5352/86881	0.71 (0.66 to 0.76)	<0.001
Standing SBP≥140	26518/66859	45939/46348	0.31 (0.30 to 0.33)	<0.001
**Placebo-Controlled Trials, N=12,577**				
ΔSBP≥20 or ΔDBP≥10	11233/76633	12103/68225	0.88 (0.83 to 0.93)	<0.001
ΔSBP≥20	1488/86613	1713/78858	0.84 (0.75 to 0.94)	0.004
ΔDBP≥10	10529/77445	11337/69290	0.88 (0.83 to 0.94)	<0.001
ΔSBP≥20 & Standing SBP≥140 (recent consensus definition)	1361/86740	1663/78908	0.80 (0.71 to 0.90)	<0.001
Standing SBP≥140	50618/37517	65397/15311	0.30 (0.28 to 0.32)	<0.001
**All Trials, N=31,124**				
ΔSBP≥20 or ΔDBP≥10	30894/149857	31967/140349	0.93 (0.90 to 0.96)	<0.001
ΔSBP≥20	7053/174256	7569/165235	0.91 (0.85 to 0.96)	0.001
ΔDBP≥10	28279/152989	29009/143801	0.94 (0.90 to 0.97)	<0.001
ΔSBP≥20 & Standing SBP≥140 (recent consensus definition)	5562/175747	7015/165789	0.73 (0.69 to 0.78)	<0.001
Standing SBP≥140	77136/104376	111336/61659	0.31 (0.30 to 0.32)	<0.001

All numbers used in definition are in units of mm Hg.
Abbreviations: BP, blood pressure; CI, confidence interval; DBP, diastolic blood pressure; OR, odds ratio; SBP, systolic blood pressure
Effects were determined via generalized estimating equations using a binomial family logit link with a robust variance estimator with adjustment for study.

Supplementary Table I. Subgroup analyses, using new consensus definition (all nine trials)

	N of Pts	N of Visits	OR (95% CI)	*P*	*P*-Interaction
**Age**					
≤ 75 years	23298	252864	0.70 (0.65 to 0.75)	<0.001	0.91
>75 years	7822	62559	0.69 (0.61 to 0.79)	<0.001	
**Sex**					
Men	16365	168933	0.66 (0.60 to 0.72)	<0.001	0.04
Women	14759	146564	0.75 (0.68 to 0.82)	<0.001	
**Race**					
Non-Black	17833	180418	0.67 (0.62 to 0.73)	<0.001	0.19
Black	6292	91018	0.74 (0.66 to 0.83)	<0.001	
**Pre-randomization SBP ≥130 or DBP ≥80 mm Hg***					
No	3669	31396	0.65 (0.56 to 0.77)	<0.001	0.34
Yes	27327	281881	0.71 (0.66 to 0.76)	<0.001	
**Diabetes**					
No	23446	269158	0.67 (0.63 to 0.72)	<0.001	0.05
Yes	7675	46320	0.78 (0.69 to 0.89)	<0.001	
**Prior Stroke**					
No	22513	198317	0.66 (0.61 to 0.71)	<0.001	0.003
Yes	3320	61224	0.81 (0.72 to 0.91)	0.001	
**Estimated GFR**					
≥ 60 mL/min per 1.73 m^2	21959	199587	0.70 (0.65 to 0.75)	<0.001	0.97
< 60 mL/min per 1.73 m^2	8583	106563	0.70 (0.62 to 0.79)	<0.001	
**Body Mass Index**					
<30 kg/m^2	19849	205648	0.73 (0.67 to 0.80)	<0.001	0.10
≥30 kg/m^2	11091	108118	0.66 (0.60 to 0.72)	<0.001	
**History of CVD**					
No	25548	237618	0.71 (0.66 to 0.76)	<0.001	0.09
Yes	4473	28849	0.61 (0.51 to 0.72)	<0.001	
**Standing SBP Just Prior Randomization***					
<140 mm Hg	26842	285204	0.70 (0.65 to 0.74)	<0.001	0.07
≥140 mm Hg	511	6153	1.20 (0.69 to 2.08)	0.53	
**Pre-randomization OHTN *(consensus definition)****					
No	26451	281482	0.69 (0.65 to 0.74)	<0.001	0.86
Yes	863	9139	0.70 (0.57 to 0.88)	0.002	

The consensus definition for orthostatic hypertension combines an orthostatic change in systolic blood pressure of at least 20 mm Hg with a standing systolic blood pressure of ≥140 mm Hg. Effects were determined via generalized estimating equations using a binomial family logit link with a robust variance estimator in strata of baseline covariates with adjustment for study. Models were restricted to follow-up visits and included an interaction term between the low

goal assignment and the stratum of interest.
*Based on visit in closest temporal proximity to randomization.
Abbreviations: CI, confidence interval; CVD, cardiovascular disease; DBP, diastolic blood pressure; GFR, glomerular filtration rate; OHTN, orthostatic hypertension; OR, odds ratio; SBP, systolic blood pressure

Supplementary Table J. Effect of more intensive treatment on orthostatic hypertension, stratified by pre-specified subgroups, truncated at the 0.1st and 99.9th percentiles (all nine trials)

	N of Pts	N of Visits	OR (95% CI)	*P*	*P*-Interaction
**Age**					
≤ 75 years	23289	251430	0.90 (0.86 to 0.93)	<0.001	0.73
>75 years	7819	62111	0.88 (0.82 to 0.95)	0.001	
**Sex**					
Men	16359	167992	0.92 (0.88 to 0.97)	0.001	0.05
Women	14753	145623	0.86 (0.82 to 0.91)	<0.001	
**Race**					
Non-Black	17827	179562	0.86 (0.83 to 0.90)	<0.001	0.002
Black	6289	90117	0.97 (0.91 to 1.04)	0.39	
**Pre-randomization SBP ≥130 or DBP ≥80 mm Hg***					
No	3665	31229	0.95 (0.86 to 1.04)	0.23	0.20
Yes	27319	280198	0.89 (0.86 to 0.92)	<0.001	
**Diabetes**					
No	23440	267526	0.88 (0.85 to 0.92)	<0.001	0.07
Yes	7669	46070	0.96 (0.88 to 1.03)	0.25	
**Prior Stroke**					
No	22505	197325	0.87 (0.83 to 0.90)	<0.001	0.05
Yes	3320	61006	0.95 (0.87 to 1.03)	0.21	
**Estimated GFR**					
≥ 60 mL/min per 1.73 m^2	21931	198555	0.89 (0.86 to 0.93)	<0.001	0.67
< 60 mL/min per 1.73 m^2	8566	105252	0.91 (0.85 to 0.97)	0.003	
**Body Mass Index**					
<30 kg/m^2	19808	204137	0.89 (0.85 to 0.93)	<0.001	0.83
≥30 kg/m^2	11056	107063	0.90 (0.85 to 0.95)	<0.001	
**History of CVD**					
No	25541	236557	0.88 (0.85 to 0.92)	<0.001	0.64
Yes	4468	28687	0.90 (0.82 to 0.99)	0.03	
**Standing SBP Just Prior Randomization**					
<140 mm Hg	26808	283159	0.89 (0.86 to 0.92)	<0.001	0.82
≥140 mm Hg	491	5724	0.91 (0.70 to 1.19)	0.49	
**Pre-randomization OHTN (*primary definition*)**					
No	22672	235334	0.89 (0.86 to 0.93)	<0.001	0.25
Yes	4523	51852	0.94 (0.87 to 1.00)	0.07	

Effects were determined via generalized estimating equations using a binomial family logit link with a robust variance estimator in strata of baseline covariates with adjustment for study. Models were restricted to follow-up visits and included an interaction term between the low goal assignment and the stratum of interest.

*Based on visit in closest temporal proximity to randomization.

Abbreviations: CI, confidence interval; CVD, cardiovascular disease; DBP, diastolic blood pressure; GFR, glomerular filtration rate; OHTN, orthostatic hypertension; OR, odds ratio; SBP, systolic blood pressure
