# Supplementary material for: Effects of intensive blood pressure treatment on orthostatic hypertension: individual level meta-analysis
Source: BMJ. 2025 Mar 25;388:e080507. doi: 10.1136/bmj-2024-080507 (PMC11934097; doi:10.1136/bmj-2024-080507)
Supplement: Supplementary file 2 — Web appendix: Supplementary figures and tables [file jurs080507.ww2.pdf]

Supplementary Methods

Supplement Methods 1. Updated search strategy

Supplement Methods 2. Analytic code

Supplement Methods 1. Updated search strategy.

Current update performed on November 13, 2023.

Medline / PubMed (National Library of Medicine, NCBI)

372 records 13 November 2023

("Hypertension"[Mesh:NoExp] OR "Essential Hypertension"[Mesh] OR "Prehypertension"[Mesh] OR hypertension[tiab] OR high blood pressure[tiab] OR prehypertension[tiab])

AND

("Hypertension/drug therapy"[Mesh] OR "Antihypertensive Agents" [Pharmacological Action] OR "Antihypertensive Agents"[Mesh] OR anti hypertensive*[tiab] OR antihypertensive*[tiab] OR blood pressure lowering[tiab] OR BP lowering[tiab] OR drug therapy[tiab] OR acetazolamide[tiab] OR amiloride[tiab] OR amlodipine[tiab] OR atenolol[tiab] OR benazepril[tiab] OR bisoprolol[tiab] OR bumetanide[tiab] OR candesartan[tiab] OR captopril[tiab] OR carvedilol[tiab] OR chlorthalidone[tiab] OR clonidine[tiab] OR diltiazem[tiab] OR doxazosin[tiab] OR ethacrynic acid[tiab] OR enalapril[tiab] OR felodipine [tiab] OR fosinopril[tiab] OR furosemide[tiab] OR hydralazine[tiab] OR hydrochlorothiazide[tiab] OR indapamide[tiab] OR irbesartan[tiab] OR lisinopril[tiab] OR losartan[tiab] OR metoprolol[tiab] OR metolazone [tiab] OR minoxidil[tiab] OR moexipril[tiab] OR nadolol[tiab] OR nebivolol[tiab] OR nifedipine[tiab] OR olmesartan[tiab] OR perindopril[tiab] OR pindolol[tiab] OR prazosin[tiab] OR propranolol[tiab] OR quinapril[tiab] OR ramipril[tiab] OR spironolactone[tiab] OR terazosin[tiab] OR torsemide[tiab] OR telmisartan[tiab] OR timolol[tiab] OR trandolapril[tiab] OR triamterene[tiab] OR verapamil[tiab])

AND

("Orthostatic Intolerance"[Mesh:NoExp] OR "Hypotension, Orthostatic"[Mesh] OR "Syncope, Vasovagal"[Mesh] OR orthostatic hypotension[tiab] OR orthostatic intolerance[tiab] OR postural hypotension[tiab] OR standing blood pressure[tiab] OR standing hypotension [tiab] OR syncope[tiab])

AND

("Randomized Controlled Trials as Topic"[Mesh] OR "Randomized Controlled Trial" [Publication Type] OR "Random Allocation"[Mesh] OR "Placebos"[Mesh] OR random*[tiab] OR placebo[tiab])

Embase (Elsevier, Embase-com)

Advanced Search

Remove mapping options

Source: Embase (1974-)

Restrict to Publication Types: Article, Conference Paper

775 records 13 November 2023

#1)

'hypertension'/de OR 'borderline hypertension'/de OR 'diabetic hypertension'/de OR 'essential hypertension'/de OR 'orthostatic hypertension'/de OR 'prehypertension'/de OR 'systolic hypertension'/de OR hypertension:ab,ti OR 'high blood pressure':ab,ti OR prehypertension:ab,ti

#2)

'antihypertensive agent'/exp/mj OR 'anti hypertensive*':ab,ti OR antihypertensive*:ab,ti OR 'blood pressure lowering':ab,ti OR 'BP lowering':ab,ti OR acetazolamide:ab,ti OR amiloride:ab,ti OR amlodipine:ab,ti OR atenolol:ab,ti OR benazepril:ab,ti OR bisoprolol:ab,ti OR bumetanide:ab,ti OR candesartan:ab,ti OR captopril:ab,ti OR carvedilol:ab,ti OR chlorthalidone:ab,ti OR clonidine:ab,ti OR diltiazem:ab,ti OR doxazosin:ab,ti OR 'ethacrynic acid':ab,ti OR enalapril:ab,ti OR felodipine:ab,ti OR fosinopril:ab,ti OR furosemide:ab,ti OR hydralazine:ab,ti OR hydrochlorothiazide:ab,ti OR indapamide:ab,ti OR irbesartan:ab,ti OR lisinopril:ab,ti OR losartan:ab,ti OR metoprolol:ab,ti OR metolazone:ab,ti OR minoxidil:ab,ti OR moexipril:ab,ti OR nadolol:ab,ti OR nebivolol:ab,ti OR nifedipine:ab,ti OR olmesartan:ab,ti OR perindopril:ab,ti OR pindolol:ab,ti OR prazosin:ab,ti OR propranolol:ab,ti OR quinapril:ab,ti OR ramipril:ab,ti OR spironolactone:ab,ti OR terazosin:ab,ti OR torsemide:ab,ti OR telmisartan:ab,ti OR timolol:ab,ti OR trandolapril:ab,ti OR triamterene:ab,ti OR verapamil:ab,ti

#3)

'orthostatic intolerance'/de OR 'orthostatic hypotension'/de OR 'faintness'/de OR 'orthostatic hypotension':ab,ti OR 'orthostatic intolerance':ab,ti OR 'postural hypotension':ab,ti OR 'standing blood pressure':ab,ti OR 'standing hypotension':ab,ti OR syncope:ab,ti

#4)

'randomized controlled trial'/exp OR 'randomized controlled trial (topic)'/de OR 'randomization'/exp OR 'placebo'/de OR random*:ab,ti OR placebo:ab,ti

(#1 AND #2 AND #3 AND #4)

Cochrane Central Register of Controlled Trials (Cochrane Library, Wiley)

488 records 13 November 2023

Advanced Search

Search Limits: remove Search Word Variations

search in Title Abstract Keywords:

hypertension OR "high blood pressure" OR prehypertension

AND

"anti hypertensive" OR "anti hypertensives" OR antihypertensive* OR "blood pressure lowering" OR "BP lowering" OR acetazolamide OR amiloride OR amlodipine OR atenolol OR benazepril OR bisoprolol OR bumetanide OR candesartan OR captopril OR carvedilol OR chlorthalidone OR clonidine OR diltiazem OR doxazosin OR “ethacrynic acid” OR enalapril OR felodipine OR fosinopril OR furosemide OR hydralazine OR hydrochlorothiazide OR indapamide OR irbesartan OR lisinopril OR losartan OR metoprolol OR metolazone OR minoxidil OR moexipril OR nadolol OR nebivolol OR nifedipine OR olmesartan OR perindopril OR pindolol OR prazosin OR propranolol OR quinapril OR ramipril OR spironolactone OR

terazosin OR torsemide OR telmisartan OR timolol OR trandolapril OR triamterene OR verapamil

AND

"orthostatic hypotension" OR "orthostatic intolerance" OR "postural hypotension" OR "standing blood pressure" OR "standing hypotension" OR syncope

TOTAL RETRIEVAL

Retrieval by database:

372 PubMed

775 Embase

488 Central

44 New Unique Records 13 November 2023

Supplement Methods 2. Analytic Code

***January 30, 2025**
***Effects of Intensive Blood Pressure Treatment on Orthostatic Hypertension: An Individual Level**
***Meta-analysis**
***Juraschek et al**

**Note: This code is intended to be run with Stata version 15.1.*

```
****************************************************
************Datasets referenced***********************
****************************************************
```

**Use dataset_dummy.dta*

**Note: This dataset is a "dummy dataset" intended to demonstrate our analytic code. The data was masked in accordance with our data use requirements, which do not permit public posting of data.*

**Please change to your local directory to run this code*

```
******************************************************************************
**********Table 2. Baseline Characteristics*****************************************
***& Supplement Table ST4  - Characteristics comparison by missing orthostatic hypertension***
******************************************************************************
```

**Annotation: This code was used to generate results included in Table 2 and Supplement Table ST4*
**Use dataset_dummy.dta*

```
cd "\Users\sjurasch\Documents\Mukamal\OH Metaanalysis\Data_files" //chance to your local directory
use dataset_dummy.dta, clear

*Overall
quietly {
foreach j in age bl_sit_sbp_v2 bl_sit_dbp_v2 bl_std_sbp_v2 bl_std_dbp_v2 bl_diff_sbp_v2
bl_diff_dbp_v2 bl_gfr_v2 bmi_v2 {
sum `j' if visit_order==1 & has_fu_v2==1
noi di "`j'" "|" r(N) "|" %3.1f r(mean) " (" %3.1f r(sd) ")"
}
}

global categ female black age75plus diabetes stroke ckd_v2 obese_v2 cvd high_bl_std_sbp_v2
bl_aOH_v2 bl_aOHTN_v2 bl_OHTN_wstd_v2
foreach j in $categ {
quietly {
prop `j' if visit_order==1 & has_fu_v2==1
matrix n=e(_N)
}
noi di "`j'" "|" e(N) "|" %3.1f _b[`j':1]*100
```

```
}

*By baseline OHTN status
quietly {
forvalues o=0/1 {
noi di "OHTN:`o'"
foreach j in age bl_sit_sbp_v2 bl_sit_dbp_v2 bl_std_sbp_v2 bl_std_dbp_v2 bl_diff_sbp_v2
bl_diff_dbp_v2 bl_gfr_v2 bmi_v2 {
sum `j' if visit_order==1 & has_fu_v2==1 & bl_aOHTN_v2==`o'
noi di "`j'" "|" r(N) "|" %3.1f r(mean) " (" %3.1f r(sd) ")"
}

global categ female black age75plus diabetes stroke ckd_v2 obese_v2 cvd high_bl_std_sbp_v2
bl_aOH_v2
foreach j in $categ {
quietly {
prop `j' if visit_order==1 & has_fu_v2==1 & bl_aOHTN_v2==`o'
matrix n=e(_N)
}
noi di "`j'" "|" e(N) "|" %3.1f _b[`j':1]*100
}
}
}

*By trial type - BP treatment
quietly {
foreach j in age bl_sit_sbp_v2 bl_sit_dbp_v2 bl_std_sbp_v2 bl_std_dbp_v2 bl_diff_sbp_v2
bl_diff_dbp_v2 bl_gfr_v2 bmi_v2 {
sum `j' if visit_order==1 & has_fu_v2==1 & study<=5
noi di "`j'" "|" r(N) "|" %3.1f r(mean) " (" %3.1f r(sd) ")"
}

global categ female black age75plus diabetes stroke ckd_v2 obese_v2 cvd high_bl_std_sbp_v2
bl_aOH_v2 bl_aOHTN_v2 bl_OHTN_wstd_v2
foreach j in $categ {
quietly {
prop `j' if visit_order==1 & has_fu_v2==1 & study<=5
matrix n=e(_N)
}
noi di "`j'" "|" e(N) "|" %3.1f _b[`j':1]*100
}
}

*By trial type - Placebo-controlled
quietly {
foreach j in age bl_sit_sbp_v2 bl_sit_dbp_v2 bl_std_sbp_v2 bl_std_dbp_v2 bl_diff_sbp_v2
bl_diff_dbp_v2 bl_gfr_v2 bmi_v2 {
sum `j' if visit_order==1 & has_fu_v2==1 & study>5 & study<=9
```

```
noi di "`j'" "|" r(N) "|" %3.1f r(mean) " (" %3.1f r(sd) ")"
}

global categ female black age75plus diabetes stroke ckd_v2 obese_v2 cvd high_bl_std_sbp_v2
bl_aOH_v2 bl_aOHTN_v2 bl_OHTN_wstd_v2
foreach j in $categ {
quietly {
prop `j' if visit_order==1 & has_fu_v2==1 & study>5 & study<=9
matrix n=e(_N)
}
noi di "`j'" "|" e(N) "|" %3.1f _b[`j':1]*100
}
}


*By missing baseline orthostatic hypertension status
*with bl_aOHTN_v2
quietly {
foreach j in age bl_sit_sbp_v2 bl_sit_dbp_v2 bl_std_sbp_v2 bl_std_dbp_v2 bl_diff_sbp_v2
bl_diff_dbp_v2 bl_gfr_v2 bmi_v2 {
sum `j' if visit_order==1 & has_fu_v2==1 & bl_aOHTN_v2!=.
noi di "`j'" "|" r(N) "|" %3.1f r(mean) " (" %3.1f r(sd) ")"
}
}

global categ female black age75plus diabetes stroke ckd_v2 obese_v2 cvd high_bl_std_sbp_v2
bl_aOH_v2 bl_aOHTN_v2 bl_OHTN_wstd_v2
foreach j in $categ {
quietly {
prop `j' if visit_order==1 & has_fu_v2==1 & bl_aOHTN_v2!=.
matrix n=e(_N)
}
noi di "`j'" "|" e(N) "|" %3.1f _b[`j':1]*100
}

*without bl_aOHTN_v2
quietly {
foreach j in age bl_sit_sbp_v2 bl_sit_dbp_v2 bl_std_sbp_v2 bl_std_dbp_v2 bl_diff_sbp_v2
bl_diff_dbp_v2 bl_gfr_v2 bmi_v2 {
sum `j' if visit_order==1 & has_fu_v2==1 & bl_aOHTN_v2==.
noi di "`j'" "|" r(N) "|" %3.1f r(mean) " (" %3.1f r(sd) ")"
}
}

global categ female black age75plus diabetes stroke ckd_v2 obese_v2 cvd
foreach j in $categ {
quietly {
prop `j' if visit_order==1 & has_fu_v2==1 & bl_aOHTN_v2==.
```

```
matrix n=e(_N)
}
noi di "`j'" "|" e(N) "|" %3.1f _b[`j':1]*100
}

clear
```

```
*********************************************************************
***********Figure 1. Prevalence Figure – primary definition******************
*********& Supplement Figure SF7 – Consensus definition*******************
********************************************************************
*Annotation: This code was used to generate results included in Figure 1 and Supplement Figure SF7
*Use dataset_dummy.dta

use dataset_dummy.dta, clear

gen study_month=round(time_to_rz/30,1)
gen study_yrs=round(time_to_rz/365.25,1)

gen study_time_cat=0 if study_month<=0 & vis==0 & has_fu_v2==1
replace study_time_cat=1 if vis!=0 & study_month<=1
replace study_time_cat=2 if vis!=0 & study_month>1 & study_month<=6
replace study_time_cat=3 if vis!=0 & study_month>6 & study_month<=12
replace study_time_cat=4 if vis!=0 & study_month>12 & study_month<=24
replace study_time_cat=5 if vis!=0 & study_month>24 & study_month<=36
replace study_time_cat=6 if vis!=0 & study_month>36 & study_month<=48
replace study_time_cat=7 if vis!=0 & study_month>48

gen study_time_cat2=0 if study_time_cat==0
replace study_time_cat2=1 if study_time_cat!=0 & study_time_cat!=.
tab study_time_cat2 study_time_cat

*Tabulation – total visits by time period by assignment
tab aOHTN_v2 study_time_cat if study<=9 & a_group_v2==1 & low_goal==0
tab aOHTN_v2 study_time_cat if study<=9 & a_group_v2==1 & low_goal==1

*number of people contributing by time period
bys study_time_cat newid: gen nvals_tmp=_n==1
bys study_time_cat newid: egen nvals=max(nvals_tmp)
bys study_time_cat newid: egen order_sq=seq() if a_group_v2==1
forvalues i=0/7 {
codebook newid if study_time_cat==`i' & study<=9 & a_group_v2==1 & low_goal==1
}
tab nvals study_time_cat if order_sq==1 & study<=9 & a_group_v2==1 & low_goal==1
forvalues i=0/7 {
codebook newid if study_time_cat==`i' & study<=9 & a_group_v2==1 & low_goal==0
}
tab nvals study_time_cat if order_sq==1 & study<=9 & a_group_v2==1 & low_goal==0

*number at risk and percent with orthostatic hypertension, horizontal orientation - by assignment
bys study_time_cat newid: egen any_aOHTN_v2=max(aOHTN_v2)

quietly {
forvalues i=0/1 {
noi di "low_goal=`i'"
```

```
forvalues j=0/7 {
prop any_aOHTN_v2 if study_time_cat==`j' & order_sq==1 & study<=9 & a_group_v2==1 &
low_goal==`i' & nvals==1
matrix n=e(_N)
sca n`j'=e(N)
sca cat`j'=_b[any_aOHTN_v2 :1]*100
}
noi di "0|1|2|3|4|5|6|7"
noi di "`=scalar(n0)' (" %3.1f `=scalar(cat0)' ") | `=scalar(n1)' (" %3.1f `=scalar(cat1)' ") | `=scalar(n2)' ("
%3.1f `=scalar(cat2)' ///
") | `=scalar(n3)' (" %3.1f `=scalar(cat3)' ") | `=scalar(n4)' (" %3.1f `=scalar(cat4)' ") | `=scalar(n5)' (" %3.1f
`=scalar(cat5)' ///
") | `=scalar(n6)' (" %3.1f `=scalar(cat6)' ") | `=scalar(n7)' (" %3.1f `=scalar(cat7)' ")"
}
}

clear

*Data for Figure – primary definition
*Use dataset_dummy.dta

use dataset_dummy.dta, clear

gen study_month=round(time_to_rz/30,1)
gen study_yrs=round(time_to_rz/365.25,1)

gen study_time_cat=0 if study_month<=0 & vis==0 & has_fu_v2==1
replace study_time_cat=1 if vis!=0 & study_month<=1
replace study_time_cat=2 if vis!=0 & study_month>1 & study_month<=6
replace study_time_cat=3 if vis!=0 & study_month>6 & study_month<=12
replace study_time_cat=4 if vis!=0 & study_month>12 & study_month<=24
replace study_time_cat=5 if vis!=0 & study_month>24 & study_month<=36
replace study_time_cat=6 if vis!=0 & study_month>36 & study_month<=48
replace study_time_cat=7 if vis!=0 & study_month>48

gen study_time_cat2=0 if study_time_cat==0
replace study_time_cat2=1 if study_time_cat!=0 & study_time_cat!=.
tab study_time_cat2 study_time_cat


*Numbers
foreach s in 9 {
quietly {
xi: xtgee aOHTN_v2 i.low_goal*i.study_time_cat2 if a_group_v2==1 & study<=`s', i(newid)
family(poisson) link(log) robust
*Standard
*Baseline
lincom _cons //standard
```

```
noi di "11|" "cat0" "|" e(N_g) "|" e(N) "|" %3.1f 100*exp(r(estimate)) "|"  %3.1f 100*exp(r(lb)) "|" %3.1f
100*exp(r(ub))
*F/u
forvalues t=1/1 {
lincom _cons + _Istudy_tim_`t' //standard
noi di "11|" "cat`t'" "|" e(N_g) "|" e(N) "|" %3.1f 100*exp(r(estimate)) "|"  %3.1f 100*exp(r(lb)) "|"
%3.1f 100*exp(r(ub))
}
*Intensive
*Baseline
//noi di "Intensive"
lincom _cons + _Ilow_goal_1 // intensive
noi di "11|" "cat0" "|" e(N_g) "|" e(N) "|" %3.1f 100*exp(r(estimate)) "|"  %3.1f 100*exp(r(lb)) "|" %3.1f
100*exp(r(ub))
*F/u
forvalues t=1/1 {
lincom _cons + _Istudy_tim_`t' + _Ilow_goal_1 + _IlowXstu_1_`t' // intensive
noi di "11|" "cat`t'" "|" e(N_g) "|" e(N) "|" %3.1f 100*exp(r(estimate)) "|"  %3.1f 100*exp(r(lb)) "|"
%3.1f 100*exp(r(ub))
}
}
}

clear

*******Consensus Definition**********
*Use dataset_dummy.dta

use dataset_dummy.dta, clear

*Percentages
gen study_month=round(time_to_rz/30,1)
gen study_yrs=round(time_to_rz/365.25,1)

gen study_time_cat=0 if study_month<=0 & vis==0 & has_fu_v2==1
replace study_time_cat=1 if vis!=0 & study_month<=1
replace study_time_cat=2 if vis!=0 & study_month>1 & study_month<=6
replace study_time_cat=3 if vis!=0 & study_month>6 & study_month<=12
replace study_time_cat=4 if vis!=0 & study_month>12 & study_month<=24
replace study_time_cat=5 if vis!=0 & study_month>24 & study_month<=36
replace study_time_cat=6 if vis!=0 & study_month>36 & study_month<=48
replace study_time_cat=7 if vis!=0 & study_month>48

gen study_time_cat2=0 if study_time_cat==0
replace study_time_cat2=1 if study_time_cat!=0 & study_time_cat!=.
tab study_time_cat2 study_time_cat

*Tabulation – total visits by time period by assignment
```

```
tab OHTN_wstd_v2 study_time_cat if study<=9 & a_group_v2==1 & low_goal==0
tab OHTN_wstd_v2 study_time_cat if study<=9 & a_group_v2==1 & low_goal==1

*number of people contributing by time period
bys study_time_cat newid: gen nvals_tmp=_n==1
bys study_time_cat newid: egen nvals=max(nvals_tmp)
bys study_time_cat newid: egen order_sq=seq() if a_group_v2==1
forvalues i=0/7 {
codebook newid if study_time_cat==`i' & study<=9 & a_group_v2==1 & low_goal==1
}
tab nvals study_time_cat if order_sq==1 & study<=9 & a_group_v2==1 & low_goal==1
forvalues i=0/7 {
codebook newid if study_time_cat==`i' & study<=9 & a_group_v2==1 & low_goal==0
}
tab nvals study_time_cat if order_sq==1 & study<=9 & a_group_v2==1 & low_goal==0

*number at risk and percent with orthostatic hypertension, horizontal orientation - by assignment
bys study_time_cat newid: egen any_OHTN_wstd_v2=max(OHTN_wstd_v2)

quietly {
forvalues i=0/1 {
noi di "low_goal=`i'"
forvalues j=0/7 {
prop any_OHTN_wstd_v2 if study_time_cat==`j' & order_sq==1 & study<=9 & a_group_v2==1 &
low_goal==`i' & nvals==1
matrix n=e(_N)
sca n`j'=e(N)
sca cat`j'=_b[any_OHTN_wstd_v2 :1]*100
}
noi di "0|1|2|3|4|5|6|7"
noi di "`=scalar(n0)' (" %3.1f `=scalar(cat0)' ") | `=scalar(n1)' (" %3.1f `=scalar(cat1)' ") | `=scalar(n2)' ("
%3.1f `=scalar(cat2)' ///
") | `=scalar(n3)' (" %3.1f `=scalar(cat3)' ") | `=scalar(n4)' (" %3.1f `=scalar(cat4)' ") | `=scalar(n5)' (" %3.1f
`=scalar(cat5)' ///
") | `=scalar(n6)' (" %3.1f `=scalar(cat6)' ") | `=scalar(n7)' (" %3.1f `=scalar(cat7)' ")"
}
}

*Data for Figure – consensus definition
*Numbers
foreach s in 9 {
quietly {
xi: xtgee OHTN_wstd_v2 i.low_goal*i.study_time_cat if a_group_v2==1 & study<=`s', i(newid)
family(poisson) link(log) robust
*Standard
*Baseline
//noi di "Standard"
lincom _cons //standard
```

```
noi di "study|cat_label|n_g|n_v|proportion|ll|ul"
noi di "11|" "cat0" "|" e(N_g) "|" e(N) "|" %3.1f 100*exp(r(estimate)) "|"  %3.1f 100*exp(r(lb)) "|" %3.1f
100*exp(r(ub))
*F/u
forvalues t=1/7 {
lincom _cons + _Istudy_tim_`t' //standard
noi di "11|" "cat`t'" "|" e(N_g) "|" e(N) "|" %3.1f 100*exp(r(estimate)) "|"  %3.1f 100*exp(r(lb)) "|"
%3.1f 100*exp(r(ub))
}
*Intensive
*Baseline
//noi di "Intensive"
lincom _cons + _Ilow_goal_1 // intensive
noi di "11|" "cat0" "|" e(N_g) "|" e(N) "|" %3.1f 100*exp(r(estimate)) "|"  %3.1f 100*exp(r(lb)) "|" %3.1f
100*exp(r(ub))
*F/u
forvalues t=1/7 {
lincom _cons + _Istudy_tim_`t' + _Ilow_goal_1 + _IlowXstu_1_`t' // intensive
noi di "11|" "cat`t'" "|" e(N_g) "|" e(N) "|" %3.1f 100*exp(r(estimate)) "|"  %3.1f 100*exp(r(lb)) "|"
%3.1f 100*exp(r(ub))
}
}
}

clear



****************************************************
******************Figure 2. Forest Plot*****************
***& Supplement Figure SF8: Consensus definition forest plot***
*****& Supplement Figure SF9: Systolic change forest plot*****
****************************************************
```

**Annotation: This code was used to generate results included in Figure 2, Supplement Figure SF8, and Supplement Figure SF9*
**Use dataset_dummy.dta*

```
use dataset_dummy.dta, clear

*numbers used in figure – primary definition
quietly {
forvalues s=1/9 {
xtgee aOHTN_v2 low_goal if vis!=0 & study==`s' & a_group_v2==1, i(newid) family(binomial) link(logit)
eform robust
matrix b=e(b)
matrix V=e(V)
foreach i in 1 {
```

```
noi di e(N_g) "|" e(N) "|" %4.2f exp(b[1,`i']) " (" %-4.2f exp(b[1,`i'] - 1.96*sqrt(V[`i',`i'])) "," %5.2f
exp(b[1,`i'] + 1.96*sqrt(V[`i',`i'])) ")" "|" round(2*ttail(e(N), abs(b[1,`i'])/sqrt(V[`i',`i'])),.001)
}
}
}
*Treatment goal trials
xtgee aOHTN_v2 low_goal i.study if vis!=0 & study<=5 & a_group_v2==1, i(newid) family(binomial)
link(logit) eform robust
matrix b=e(b)
matrix V=e(V)
foreach i in 1 {
noi di e(N_g) "|" e(N) "|" %4.2f exp(b[1,`i']) " (" %-4.2f exp(b[1,`i'] - 1.96*sqrt(V[`i',`i'])) "," %5.2f
exp(b[1,`i'] + 1.96*sqrt(V[`i',`i'])) ")" "|" round(2*ttail(e(N), abs(b[1,`i'])/sqrt(V[`i',`i'])),.001)
}

*Placebo only
xtgee aOHTN_v2 low_goal i.study if vis!=0 & a_group_v2==1 & study>5 & study<=9, i(newid)
family(binomial) link(logit) eform robust
matrix b=e(b)
matrix V=e(V)
foreach i in 1 {
noi di e(N_g) "|" e(N) "|" %4.2f exp(b[1,`i']) " (" %-4.2f exp(b[1,`i'] - 1.96*sqrt(V[`i',`i'])) "," %5.2f
exp(b[1,`i'] + 1.96*sqrt(V[`i',`i'])) ")" "|" round(2*ttail(e(N), abs(b[1,`i'])/sqrt(V[`i',`i'])),.001)
}

*All nine trials
xtgee aOHTN_v2 low_goal i.study if vis!=0 & a_group_v2==1, i(newid) family(binomial) link(logit) eform
robust
matrix b=e(b)
matrix V=e(V)
foreach i in 1 {
noi di e(N_g) "|" e(N) "|" %4.2f exp(b[1,`i']) " (" %-4.2f exp(b[1,`i'] - 1.96*sqrt(V[`i',`i'])) "," %5.2f
exp(b[1,`i'] + 1.96*sqrt(V[`i',`i'])) ")" "|" round(2*ttail(e(N), abs(b[1,`i'])/sqrt(V[`i',`i'])),.001)
}

*numbers used in figure – systolic change definition, consensus definition
*OHTN
foreach o in sOHTN_v2 OHTN_wstd_v2 {
noi di "`o'"
quietly {
forvalues s=1/9 {
xtgee `o' low_goal if vis!=0 & study==`s' & a_group_v2==1, i(newid) family(binomial) link(logit) robust
matrix b=e(b)
matrix V=e(V)
foreach i in 1 {
noi di e(N_g) "|" e(N) "|" %4.3f b[1,`i'] "|" %-4.3f b[1,`i'] - 1.96*sqrt(V[`i',`i']) "|" %5.3f b[1,`i'] +
1.96*sqrt(V[`i',`i'])
}
```

```
}
*Primary
xtgee `o' low_goal i.study if vis!=0 & study<=5 & a_group_v2==1, i(newid) family(binomial) link(logit)
robust
matrix b=e(b)
matrix V=e(V)
foreach i in 1 {
noi di e(N_g) "|" e(N) "|" %4.3f b[1,`i'] "|" %-4.3f b[1,`i'] - 1.96*sqrt(V[`i',`i']) "|" %5.3f b[1,`i'] +
1.96*sqrt(V[`i',`i'])
}
*Placebo
xtgee `o' low_goal i.study if vis!=0 & study>5 & study<=9 & a_group_v2==1, i(newid) family(binomial)
link(logit) robust
matrix b=e(b)
matrix V=e(V)
foreach i in 1 {
noi di e(N_g) "|" e(N) "|" %4.3f b[1,`i'] "|" %-4.3f b[1,`i'] - 1.96*sqrt(V[`i',`i']) "|" %5.3f b[1,`i'] +
1.96*sqrt(V[`i',`i'])
}
*Extended
xtgee `o' low_goal i.study if vis!=0 & a_group_v2==1, i(newid) family(binomial) link(logit) robust
matrix b=e(b)
matrix V=e(V)
foreach i in 1 {
noi di e(N_g) "|" e(N) "|" %4.3f b[1,`i'] "|" %-4.3f b[1,`i'] - 1.96*sqrt(V[`i',`i']) "|" %5.3f b[1,`i'] +
1.96*sqrt(V[`i',`i'])
}
}
}
clear


*****************************************************************
***************Poisson Sensitivity – Referenced in Methods*************
*****************************************************************
*Annotation: This code was used to run a Poisson sensitivity analysis references in the Methods
*Use dataset_dummy.dta

use dataset_dummy.dta, clear

xtgee aOHTN_v2 low_goal i.study if vis!=0 & a_group_v2==1, i(newid) family(poisson) link(log) eform
robust

quietly {
forvalues s=1/9 {
xtgee aOHTN_v2 low_goal if vis!=0 & study==`s' & a_group_v2==1, i(newid) family(poisson) link(log)
eform robust
matrix b=e(b)
```

```
matrix V=e(V)
foreach i in 1 {
noi di e(N_g) "|" e(N) "|" %4.2f exp(b[1,`i']) " (" %-4.2f exp(b[1,`i'] - 1.96*sqrt(V[`i',`i'])) "," %5.2f
exp(b[1,`i'] + 1.96*sqrt(V[`i',`i'])) ")" "|" round(2*ttail(e(N), abs(b[1,`i'])/sqrt(V[`i',`i'])),.001)
}
}
}

*Primary
xtgee aOHTN_v2 low_goal i.study if vis!=0 & study<=5 & a_group_v2==1, i(newid) family(poisson)
link(log) eform robust
matrix b=e(b)
matrix V=e(V)
foreach i in 1 {
noi di e(N_g) "|" e(N) "|" %4.2f exp(b[1,`i']) " (" %-4.2f exp(b[1,`i'] - 1.96*sqrt(V[`i',`i'])) "," %5.2f
exp(b[1,`i'] + 1.96*sqrt(V[`i',`i'])) ")" "|" round(2*ttail(e(N), abs(b[1,`i'])/sqrt(V[`i',`i'])),.001)
}

*Extended
xtgee aOHTN_v2 low_goal i.study if vis!=0 & a_group_v2==1, i(newid) family(poisson) link(log) eform
robust
matrix b=e(b)
matrix V=e(V)
foreach i in 1 {
noi di e(N_g) "|" e(N) "|" %4.2f exp(b[1,`i']) " (" %-4.2f exp(b[1,`i'] - 1.96*sqrt(V[`i',`i'])) "," %5.2f
exp(b[1,`i'] + 1.96*sqrt(V[`i',`i'])) ")" "|" round(2*ttail(e(N), abs(b[1,`i'])/sqrt(V[`i',`i'])),.001)
}

*Placebo only
xtgee aOHTN_v2 low_goal i.study if vis!=0 & a_group_v2==1 & study>5 & study<=9, i(newid)
family(poisson) link(log) eform robust
matrix b=e(b)
matrix V=e(V)
foreach i in 1 {
noi di e(N_g) "|" e(N) "|" %4.2f exp(b[1,`i']) " (" %-4.2f exp(b[1,`i'] - 1.96*sqrt(V[`i',`i'])) "," %5.2f
exp(b[1,`i'] + 1.96*sqrt(V[`i',`i'])) ")" "|" round(2*ttail(e(N), abs(b[1,`i'])/sqrt(V[`i',`i'])),.001)
}

clear
```

```
**************************************************
***********Table 3. Alternate Definitions & ***************
*******Supplement ST8, using truncated percentiles*********
**************************************************
*Annotation: This code was used to generate results included in Table 3 and Supplement Table ST8
*Use dataset_dummy.dta

use dataset_dummy.dta, clear

*Secondary definitions - tabulations
quietly{
noi di "BP goal trials"
foreach e in aOHTN_v2 sOHTN_v2 dOHTN_v2 OHTN_wstd_v2 sts140 {
tab low_goal `e' if study<=5 & a_group_v2==1, matcell(x)
noi di "low_goal=1e/ne/0e/ne|`e'|" (x[2,2]) "/" (x[2,1]) "|" (x[1,2]) "/" (x[1,1])
}
noi di "Placebo trials"
foreach e in aOHTN_v2 sOHTN_v2 dOHTN_v2 OHTN_wstd_v2 sts140 {
tab low_goal `e' if study>=6 & study<=9 & a_group_v2==1, matcell(x)
noi di "low_goal=1e/ne/0e/ne|`e'|" (x[2,2]) "/" (x[2,1]) "|" (x[1,2]) "/" (x[1,1])
}
noi di "All trials"
foreach e in aOHTN_v2 sOHTN_v2 dOHTN_v2 OHTN_wstd_v2 sts140 {
tab low_goal `e' if study<=9 & a_group_v2==1, matcell(x)
noi di "low_goal=1e/ne/0e/ne|`e'|" (x[2,2]) "/" (x[2,1]) "|" (x[1,2]) "/" (x[1,1])
}
}


*BP goal
quietly {
foreach j in aOHTN_v2 sOHTN_v2 dOHTN_v2 OHTN_wstd_v2 sts140 {
xtgee `j' low_goal i.study if vis!=0 & study<=5 & a_group_v2==1, i(newid) family(binomial) link(logit)
eform robust
matrix b=e(b)
matrix V=e(V)
foreach i in 1 {
noi di "`j'|" e(N_g) "|" e(N) "|" %4.2f exp(b[1,`i']) " (" %-4.2f exp(b[1,`i'] - 1.96*sqrt(V[`i',`i'])) "," %5.2f
exp(b[1,`i'] + 1.96*sqrt(V[`i',`i'])) ")" "|" round(2*ttail(e(N), abs(b[1,`i'])/sqrt(V[`i',`i'])),.001)
}
}
}
*Placebo only
quietly {
foreach j in aOHTN_v2 sOHTN_v2 dOHTN_v2 OHTN_wstd_v2 sts140 {
xtgee `j' low_goal i.study if vis!=0 & a_group_v2==1 & study>5 & study<=9, i(newid) family(binomial)
link(logit) eform robust
matrix b=e(b)
```

```
matrix V=e(V)
foreach i in 1 {
noi di "`j'|" e(N_g) "|" e(N) "|" %4.2f exp(b[1,`i']) " (" %-4.2f exp(b[1,`i'] - 1.96*sqrt(V[`i',`i'])) "," %5.2f
exp(b[1,`i'] + 1.96*sqrt(V[`i',`i'])) ")" "|" round(2*ttail(e(N), abs(b[1,`i'])/sqrt(V[`i',`i'])),.001)
}
}
}
*Extended (all nine)
quietly {
foreach j in aOHTN_v2 sOHTN_v2 dOHTN_v2 OHTN_wstd_v2 sts140 {
xtgee `j' low_goal i.study if vis!=0 & a_group_v2==1, i(newid) family(binomial) link(logit) eform robust
matrix b=e(b)
matrix V=e(V)
foreach i in 1 {
noi di "`j'|" e(N_g) "|" e(N) "|" %4.2f exp(b[1,`i']) " (" %-4.2f exp(b[1,`i'] - 1.96*sqrt(V[`i',`i'])) "," %5.2f
exp(b[1,`i'] + 1.96*sqrt(V[`i',`i'])) ")" "|" round(2*ttail(e(N), abs(b[1,`i'])/sqrt(V[`i',`i'])),.001)
}
}
}

*Truncated analyses
*Secondary definitions - tabulations
quietly{
noi di "BP goal trials"
foreach e in aOHTN_v3 sOHTN_v3 dOHTN_v3 OHTN_wstd_v3 sts140_v3 {
tab low_goal `e' if study<=5 & a_group_v2==1, matcell(x)
noi di "low_goal=1e/ne/0e/ne|`e'|" (x[2,2]) "/" (x[2,1]) "|" (x[1,2]) "/" (x[1,1])
}
noi di "Placebo trials"
foreach e in aOHTN_v3 sOHTN_v3 dOHTN_v3 OHTN_wstd_v3 sts140_v3 {
tab low_goal `e' if study>=6 & study<=9 & a_group_v2==1, matcell(x)
noi di "low_goal=1e/ne/0e/ne|`e'|" (x[2,2]) "/" (x[2,1]) "|" (x[1,2]) "/" (x[1,1])
}
noi di "All trials"
foreach e in aOHTN_v3 sOHTN_v3 dOHTN_v3 OHTN_wstd_v3 sts140_v3 {
tab low_goal `e' if study<=9 & a_group_v2==1, matcell(x)
noi di "low_goal=1e/ne/0e/ne|`e'|" (x[2,2]) "/" (x[2,1]) "|" (x[1,2]) "/" (x[1,1])
}
}

*BP goal
quietly {
foreach j in aOHTN_v3 sOHTN_v3 dOHTN_v3 OHTN_wstd_v3 sts140_v3 {
xtgee `j' low_goal i.study if vis!=0 & study<=5 & a_group_v2==1, i(newid) family(binomial) link(logit)
eform robust
matrix b=e(b)
matrix V=e(V)
foreach i in 1 {
```

```
noi di "`j'|" e(N_g) "|" e(N) "|" %4.2f exp(b[1,`i']) " (" %-4.2f exp(b[1,`i'] - 1.96*sqrt(V[`i',`i'])) "," %5.2f
exp(b[1,`i'] + 1.96*sqrt(V[`i',`i'])) ")" "|" round(2*ttail(e(N), abs(b[1,`i'])/sqrt(V[`i',`i'])),.001)
}
}
}
*Placebo only
quietly {
foreach j in aOHTN_v3 sOHTN_v3 dOHTN_v3 OHTN_wstd_v3 sts140_v3 {
xtgee `j' low_goal i.study if vis!=0 & a_group_v2==1 & study>5 & study<=9, i(newid) family(binomial)
link(logit) eform robust
matrix b=e(b)
matrix V=e(V)
foreach i in 1 {
noi di "`j'|" e(N_g) "|" e(N) "|" %4.2f exp(b[1,`i']) " (" %-4.2f exp(b[1,`i'] - 1.96*sqrt(V[`i',`i'])) "," %5.2f
exp(b[1,`i'] + 1.96*sqrt(V[`i',`i'])) ")" "|" round(2*ttail(e(N), abs(b[1,`i'])/sqrt(V[`i',`i'])),.001)
}
}
}
*Extended (all nine)
quietly {
foreach j in aOHTN_v3 sOHTN_v3 dOHTN_v3 OHTN_wstd_v3 sts140_v3 {
xtgee `j' low_goal i.study if vis!=0 & a_group_v2==1, i(newid) family(binomial) link(logit) eform robust
matrix b=e(b)
matrix V=e(V)
foreach i in 1 {
noi di "`j'|" e(N_g) "|" e(N) "|" %4.2f exp(b[1,`i']) " (" %-4.2f exp(b[1,`i'] - 1.96*sqrt(V[`i',`i'])) "," %5.2f
exp(b[1,`i'] + 1.96*sqrt(V[`i',`i'])) ")" "|" round(2*ttail(e(N), abs(b[1,`i'])/sqrt(V[`i',`i'])),.001)
}
}
}


clear
```

```
***************************************************************
***********Table 4. Stratified Analysis********************************
***********Supplement Table ST9. Consensus definition******************
********& Supplement Table ST10 Truncated percentiles******************
***************************************************************
```

**Annotation: This code was used to generate results included in Table 4, Supplement Figure ST9, and Supplement Table ST10*
**Use dataset_dummy.dta*

```
use dataset_dummy.dta, clear

gen stratum1=age75plus
gen stratum2=female
gen stratum3=black
gen stratum4=bl_htn
gen stratum5=diabetes
gen stratum6=stroke
gen stratum7=ckd_v2
gen stratum8=obese_v2
gen stratum9=cvd
gen stratum10=high_bl_std_sbp_v2
gen stratum11=bl_aOHTN_v2

*All studies
*OHTN
quietly {
foreach s in age75plus female black bl_htn diabetes stroke ckd_v2 obese_v2 cvd high_bl_std_sbp_v2
bl_aOHTN_v2 {
noi di "stratum=`s'"
forvalues t=0/1 {
xtgee aOHTN_v2 low_goal if vis!=0 & `s'==`t' & a_group_v2==1, i(newid) family(binomial) link(logit)
eform robust
matrix b=e(b)
matrix V=e(V)
foreach i in 1 {
noi di e(N_g) "|" e(N) "|" %4.2f exp(b[1,`i']) " (" %-4.2f exp(b[1,`i'] - 1.96*sqrt(V[`i',`i'])) "," %5.2f
exp(b[1,`i'] + 1.96*sqrt(V[`i',`i'])) ")" "|" round(2*ttail(e(N), abs(b[1,`i'])/sqrt(V[`i',`i'])),.001)
}
}
}
}

*P-interaction
quietly {
foreach s in age75plus female black bl_htn diabetes stroke ckd_v2 obese_v2 cvd high_bl_std_sbp_v2
bl_aOHTN_v2 {
noi di ""
noi di "stratum=`s'"
```

```
xi: xtgee aOHTN_v2 i.low_goal*i.`s' if vis!=0 & a_group_v2==1, i(newid) family(binomial) link(logit) eform
robust
matrix b=e(b)
matrix V=e(V)
foreach i in 3 {
noi di e(N_g) "|" e(N) /*"|" %4.2f exp(b[1,`i']) " (" %-4.2f exp(b[1,`i'] - 1.96*sqrt(V[`i',`i'])) "," %5.2f
exp(b[1,`i'] + 1.96*sqrt(V[`i',`i'])) ")"*/ "|" round(2*ttail(e(N), abs(b[1,`i'])/sqrt(V[`i',`i'])),.001)
}
}
}

clear

****Stratified analysis - consensus****
*Use dataset_dummy.dta

use dataset_dummy.dta, clear

gen stratum1=age75plus
gen stratum2=female
gen stratum3=black
gen stratum4=bl_htn
gen stratum5=diabetes
gen stratum6=stroke
gen stratum7=ckd_v2
gen stratum8=obese_v2
gen stratum9=cvd
gen stratum10=high_bl_std_sbp_v2
gen stratum11=bl_OHTN_wstd_v2

*All studies
*OHTN
quietly {
foreach s in age75plus female black bl_htn diabetes stroke ckd_v2 obese_v2 cvd high_bl_std_sbp_v2
bl_OHTN_wstd_v2 {
noi di "stratum=`s'"
forvalues t=0/1 {
xtgee OHTN_wstd_v2 low_goal if vis!=0 & `s'==`t' & a_group_v2==1, i(newid) family(binomial) link(logit)
eform robust
matrix b=e(b)
matrix V=e(V)
foreach i in 1 {
noi di e(N_g) "|" e(N) "|" %4.2f exp(b[1,`i']) " (" %-4.2f exp(b[1,`i'] - 1.96*sqrt(V[`i',`i'])) "," %5.2f
exp(b[1,`i'] + 1.96*sqrt(V[`i',`i'])) ")" "|" round(2*ttail(e(N), abs(b[1,`i'])/sqrt(V[`i',`i'])),.001)
}
}
}
}
```

```
*P-interaction
quietly {
foreach s in age75plus female black bl_htn diabetes stroke ckd_v2 obese_v2 cvd high_bl_std_sbp_v2
bl_OHTN_wstd_v2 {
noi di ""
noi di "stratum=`s'"
xi: xtgee OHTN_wstd_v2 i.low_goal*i.`s' if vis!=0 & a_group_v2==1, i(newid) family(binomial) link(logit)
eform robust
matrix b=e(b)
matrix V=e(V)
foreach i in 3 {
noi di e(N_g) "|" e(N) /*"|" %4.2f exp(b[1,`i']) " (" %-4.2f exp(b[1,`i'] - 1.96*sqrt(V[`i',`i'])) "," %5.2f
exp(b[1,`i'] + 1.96*sqrt(V[`i',`i'])) ")"*/ "|" round(2*ttail(e(N), abs(b[1,`i'])/sqrt(V[`i',`i'])),.001)
}
}
}

clear

****Stratified analysis - truncated at 0.1/99.9****
```
**Use dataset_dummy.dta*

```
use dataset_dummy.dta, clear

gen stratum1=age75plus
gen stratum2=female
gen stratum3=black
gen stratum4=bl_htn
gen stratum5=diabetes
gen stratum6=stroke
gen stratum7=ckd_v3
gen stratum8=obese_v3
gen stratum9=cvd
gen stratum10=high_bl_std_sbp_v3
gen stratum11=bl_aOHTN_v3

*All studies
*OHTN
quietly {
foreach s in age75plus female black bl_htn diabetes stroke ckd_v3 obese_v3 cvd high_bl_std_sbp_v3
bl_aOHTN_v3 {
noi di "stratum=`s'"
forvalues t=0/1 {
xtgee aOHTN_v3 low_goal if vis!=0 & `s'==`t' & a_group_v2==1, i(newid) family(binomial) link(logit)
eform robust
matrix b=e(b)
matrix V=e(V)
```

```
foreach i in 1 {
noi di e(N_g) "|" e(N) "|" %4.2f exp(b[1,`i']) " (" %-4.2f exp(b[1,`i'] - 1.96*sqrt(V[`i',`i'])) "," %5.2f
exp(b[1,`i'] + 1.96*sqrt(V[`i',`i'])) ")" "|" round(2*ttail(e(N), abs(b[1,`i'])/sqrt(V[`i',`i'])),.001)
}
}
}
}

*P-interaction
quietly {
foreach s in age75plus female black bl_htn diabetes stroke ckd_v3 obese_v3 cvd high_bl_std_sbp_v3
bl_aOHTN_v3 {
noi di ""
noi di "stratum=`s'"
xi: xtgee aOHTN_v3 i.low_goal*i.`s' if vis!=0 & a_group_v2==1, i(newid) family(binomial) link(logit) eform
robust
matrix b=e(b)
matrix V=e(V)
foreach i in 3 {
noi di e(N_g) "|" e(N) /*"|" %4.2f exp(b[1,`i']) " (" %-4.2f exp(b[1,`i'] - 1.96*sqrt(V[`i',`i'])) "," %5.2f
exp(b[1,`i'] + 1.96*sqrt(V[`i',`i'])) ")"*/ "|" round(2*ttail(e(N), abs(b[1,`i'])/sqrt(V[`i',`i'])),.001)
}
}
}


clear
```

```
*************************************************************
***********Supplement Table ST2 & ST3. Truncations******************
************************************************************
*Annotation: This code was used to generate results included in Supplement Table ST2 and ST3
*Use dataset_dummy.dta

use dataset_dummy.dta, clear

*table of extremes at .01 and 99.99 percentiles
quietly {
foreach i in bl_gfr bmi ///
bl_sit_sbp bl_sit_dbp bl_std_sbp bl_std_dbp ///
sit_sbp sit_dbp std_sbp std_dbp {
centile `i', centile(.01 99.99)
noi di "`i'|" %3.2f r(c_1) "|" %3.2f r(c_2)
}
}

*table of extremes at .1 and 99.9 percentiles
quietly {
foreach i in bl_gfr bmi ///
bl_sit_sbp bl_sit_dbp bl_std_sbp bl_std_dbp ///
sit_sbp sit_dbp std_sbp std_dbp {
centile `i', centile(.1 99.9)
noi di "`i'|" %3.2f r(c_1) "|" %3.2f r(c_2)
}
}


clear
```

```
****************************************************************
***********Supplement Figures SF2-3. Kernel density plots****************
****************************************************************
*Annotation: This code was used to generate results included in Supplement Figures SF2-3
*Use dataset_dummy.dta

use dataset_dummy.dta, clear

*Number of visits
gen has_bl_bp_tmp=1 if vis==0 & bl_sit_sbp_v2!=. & bl_sit_dbp_v2!=. & bl_std_sbp_v2!=. &
bl_std_dbp_v2!=.
bys newid: egen has_bl_bp=min(has_bl_bp_tmp)
gen has_fv_bp_tmp=1 if vis!=0 & sit_sbp_v2!=. & sit_dbp_v2!=. & std_sbp_v2!=. & std_dbp_v2!=.
bys newid: egen has_fv_bp=min(has_fv_bp_tmp)
tab has_bl_bp has_fv_bp, missing
gen bl_kdense=1 if visit_order==1 & has_bl_bp==1 & has_fv_bp==1
gen fv_kdense=1 if has_bl_bp==1 & has_fv_bp==1 & a_group_v2==1 & vis!=0

tab low_goal bl_kdense
tab low_goal fv_kdense

*Seated systolic blood pressure
set scheme s1color
#delimit ;
twoway
(kdensity bl_sit_sbp_v2 if bl_kdense==1, bw(5) lpattern(solid) lcolor(blue) lwidth(thick))
(kdensity sit_sbp_v2 if fv_kdense==1 & low_goal==0, bw(5) lpattern(dash) lcolor(red) lwidth(thick))
(kdensity sit_sbp_v2 if fv_kdense==1 & low_goal==1, bw(5) lpattern(shortdash) lcolor(green)
lwidth(thick))
,
ysize(5)
ytitle("Probability Density", size(medium))
xtitle("Seated SBP (mm Hg)", height(7) size(medium))
xlabel(60(20)220, labsize(small) angle(horizontal) nogrid)
ylabel(0 "0.000" .005 "0.005" .01 "0.010" .015 "0.015" .02 "0.020" .025 "0.025", angle(horizontal))
plotregion(style(none))
xsize(5)
xmtick(none)
legend(off)
/*legend(label(1 "Baseline") label(2 "Low") label(3 "Standard"))
legend(order(1 2 3)) */
name(kd_sit_sbp_v2, replace)
;
#delimit cr


*Standing systolic blood pressure
set scheme s1color
```

```
#delimit ;
twoway
(kdensity bl_std_sbp_v2 if bl_kdense==1, bw(5) lpattern(solid) lcolor(blue) lwidth(thick))
(kdensity std_sbp_v2 if fv_kdense==1 & low_goal==0, bw(5) lpattern(dash) lcolor(red) lwidth(thick))
(kdensity std_sbp_v2 if fv_kdense==1 & low_goal==1, bw(5) lpattern(shortdash) lcolor(green)
lwidth(thick))
,
ysize(5)
ytitle("Probability Density", size(medium))
xtitle("Standing SBP (mm Hg)", height(7) size(medium))
xlabel(60(20)220, labsize(small) angle(horizontal) nogrid)
ylabel(0 "0.000" .005 "0.005" .01 "0.010" .015 "0.015" .02 "0.020" .025 "0.025", angle(horizontal))
plotregion(style(none))
xsize(5)
xmtick(none)
legend(off)
/*legend(label(1 "Baseline") label(2 "Low") label(3 "Standard"))
legend(order(1 2 3)) */
name(kd_std_sbp_v2, replace)
;
#delimit cr


*Difference in systolic blood pressure
set scheme s1color
#delimit ;
twoway
(kdensity bl_diff_sbp_v2 if bl_kdense==1, bw(5) lpattern(solid) lcolor(blue) lwidth(thick))
(kdensity diff_sbp_v2 if fv_kdense==1 & low_goal==0, bw(5) lpattern(dash) lcolor(red) lwidth(thick))
(kdensity diff_sbp_v2 if fv_kdense==1 & low_goal==1, bw(5) lpattern(shortdash) lcolor(green)
lwidth(thick))
,
ysize(5)
ytitle("Probability Density", size(medium))
xtitle("Postural Change in SBP (mm Hg)", height(7) size(medium))
xlabel(-80(20)80, labsize(small) angle(horizontal) nogrid)
ylabel(0 "0.000" .005 "0.005" .01 "0.010" .015 "0.015" .02 "0.020" .025 "0.025" .03 "0.030" .035 "0.035",
angle(horizontal))
plotregion(style(none))
xsize(5)
xmtick(none)
legend(off)
/*legend(label(1 "Baseline") label(2 "Intensive") label(3 "Standard"))
legend(order(1 2 3))*/
name(kd_diff_sbp_v2, replace)

;
#delimit cr
```

```
***DBP***
*Seated
set scheme s1color
#delimit ;
twoway
(kdensity bl_sit_dbp_v2 if bl_kdense==1, bw(5) lpattern(solid) lcolor(blue) lwidth(thick))
(kdensity sit_dbp_v2 if fv_kdense==1 & low_goal==0, bw(5) lpattern(dash) lcolor(red) lwidth(thick))
(kdensity sit_dbp_v2 if fv_kdense==1 & low_goal==1, bw(5) lpattern(shortdash) lcolor(green)
lwidth(thick))
,
ysize(5)
ytitle("Probability Density", size(medium))
xtitle("Seated DBP (mm Hg)", height(7) size(medium))
xlabel(20(20)160, labsize(small) angle(horizontal) nogrid)
ylabel(0 "0.000" .005 "0.005" .01 "0.010" .015 "0.015" .02 "0.020" .025 "0.025" .03 "0.030" .035 "0.035",
angle(horizontal))
plotregion(style(none))
xsize(5)
xmtick(none)
legend(off)
/*legend(label(1 "Baseline") label(2 "Low") label(3 "Standard"))
legend(order(1 2 3)) */
name(kd_sit_dbp, replace)
;
#delimit cr


*Standing
set scheme s1color
#delimit ;
twoway
(kdensity bl_std_dbp_v2 if bl_kdense==1, bw(5) lpattern(solid) lcolor(blue) lwidth(thick))
(kdensity std_dbp_v2 if fv_kdense==1 & low_goal==0, bw(5) lpattern(dash) lcolor(red) lwidth(thick))
(kdensity std_dbp_v2 if fv_kdense==1 & low_goal==1, bw(5) lpattern(shortdash) lcolor(green)
lwidth(thick))
,
ysize(5)
ytitle("Probability Density", size(medium))
xtitle("Standing DBP (mm Hg)", height(7) size(medium))
xlabel(20(20)160, labsize(small) angle(horizontal) nogrid)
ylabel(0 "0.000" .005 "0.005" .01 "0.010" .015 "0.015" .02 "0.020" .025 "0.025" .03 "0.030" .035 "0.035",
angle(horizontal))
plotregion(style(none))
xsize(5)
xmtick(none)
legend(off)
```

```
/*legend(label(1 "Baseline") label(2 "Low") label(3 "Standard"))
legend(order(1 2 3)) */
name(kd_std_dbp_v2, replace)
;
#delimit cr


*Difference
set scheme s1color
#delimit ;
twoway
(kdensity bl_diff_dbp_v2 if bl_kdense==1, bw(5) lpattern(solid) lcolor(blue) lwidth(thick))
(kdensity diff_dbp_v2 if fv_kdense==1 & low_goal==0, bw(5) lpattern(dash) lcolor(red) lwidth(thick))
(kdensity diff_dbp_v2 if fv_kdense==1 & low_goal==1, bw(5) lpattern(shortdash) lcolor(green)
lwidth(thick))
,
ysize(5)
ytitle("Probability Density", size(medium))
xtitle("Postural Change in DBP (mm Hg)", height(7) size(medium))
xlabel(-40(20)60, labsize(small) angle(horizontal) nogrid)
ylabel(0 "0.000" .005 "0.005" .01 "0.010" .015 "0.015" .02 "0.020" .025 "0.025" .03 "0.030" .035 "0.035"
.04 "0.040" .045 "0.045", angle(horizontal))
plotregion(style(none))
xsize(5)
xmtick(none)
legend(off)
/*legend(label(1 "Baseline") label(2 "Intensive") label(3 "Standard"))
legend(order(1 2 3))*/
name(kd_diff_dbp_v2, replace)

;
#delimit cr



clear
```

```
*******************************************************************
***********Supplement Figures SF4-6. Two-stage meta-analyses****************
*******************************************************************
*Annotation: This code was used to generate results included in Supplement Figures SF4-6
*Use dataset_dummy.dta

use dataset_dummy.dta, clear

putexcel set "forest_primary.xlsx", sheet("Table 1", replace) replace

putexcel A1 = "study"
putexcel B1 = "groups"
putexcel C1 = "number"
putexcel D1 = "logor"
putexcel E1 = "selogor"
putexcel F1 = "studylabel"

local count_row = 2

forvalues s=1/9 {
xtgee aOHTN_v2 low_goal if vis!=0 & study==`s' & a_group_v2==1, i(newid) family(binomial) link(logit)
robust
matrix b=e(b)
matrix V=e(V)
sca N_g=e(N_g)
sca N_m=e(N)
putexcel A`count_row' = `s'
putexcel B`count_row' = `=scalar(N_g)'
putexcel C`count_row' = `=scalar(N_m)'
lincom low_goal
putexcel D`count_row' = `r(estimate)'
putexcel E`count_row' = `r(se)'

local count_row = `count_row' + 1
}

putexcel A1 = "study"
putexcel F2 = "AASK"
putexcel F3 = "ACCORD"
putexcel F4 = "SPRINT"
putexcel F5 = "SPS3"
putexcel F6 = "UKPDS"
putexcel F7 = "HYVET"
putexcel F8 = "SHEP"
putexcel F9 = "SYST-EUR"
putexcel F10 = "TOMHS"

import excel "forest_primary.xlsx", sheet("Table 1") firstrow clear
```

```
metan logor selogor if study<=5, eform xlabel(0.5, 1, 1.5, 2, 2.5) random
metan logor selogor if study>=6 & study<=9, eform xlabel(0.5, 1, 1.5, 2, 2.5) random
metan logor selogor, eform xlabel(0.5, 1, 1.5, 2, 2.5) label(namevar=studylabel) xsize(5) ysize(5) random

metabias logor selogor if study<=5, egger
metabias logor selogor if study>=6 & study<=9, egger
metabias logor selogor, egger

metafunnel logor selogor if study<=5, xtitle("Odds ratio (log scale)", height(7)) ytitle("Standard Error",
height(7)) ///
xsize(5) ysize(5) xlabel(-.4 "-0.4" -.2 "-0.2" 0 "0.0" .2 "0.2") ylabel(.15 "0.15" .1 "0.10" .05 "0.05" 0 "0.0")
name(meta5_primary, replace)
metafunnel logor selogor if study>=6 & study<=9, xtitle("Odds ratio (log scale)", height(7))
ytitle("Standard Error", height(7)) ///
xsize(5) ysize(5) xlabel(-.4 "-0.4" -.2 "-0.2" 0 "0.0" .2 "0.2") ylabel(.15 "0.15" .1 "0.10" .05 "0.05" 0 "0.0")
name(meta5_primary, replace)
metafunnel logor selogor, xtitle("Odds ratio (log scale)", height(7)) ytitle("Standard Error", height(7)) ///
xsize(5) ysize(5) xlabel(-.4 "-0.4" -.2 "-0.2" 0 "0.0" .2 "0.2") ylabel(.15 "0.15" .1 "0.10" .05 "0.05" 0 "0.0")
name(meta9_primary, replace)

clear

*******************Consensus*******************
*Use dataset_dummy.dta

use dataset_dummy.dta, clear


putexcel set "forest_consensus.xlsx", sheet("Table 1", replace) replace

putexcel A1 = "study"
putexcel B1 = "groups"
putexcel C1 = "number"
putexcel D1 = "logor"
putexcel E1 = "selogor"
putexcel F1 = "studylabel"

local count_row = 2

forvalues s=1/9 {
xtgee OHTN_wstd_v2 low_goal if vis!=0 & study==`s' & a_group_v2==1, i(newid) family(binomial)
link(logit) robust
matrix b=e(b)
matrix V=e(V)
sca N_g=e(N_g)
sca N_m=e(N)
putexcel A`count_row' = `s'
```

```
putexcel B`count_row' = `=scalar(N_g)'
putexcel C`count_row' = `=scalar(N_m)'
lincom low_goal
putexcel D`count_row' = `r(estimate)'
putexcel E`count_row' = `r(se)'

local count_row = `count_row' + 1
}

putexcel A1 = "study"
putexcel F2 = "AASK"
putexcel F3 = "ACCORD"
putexcel F4 = "SPRINT"
putexcel F5 = "SPS3"
putexcel F6 = "UKPDS"
putexcel F7 = "HYVET"
putexcel F8 = "SHEP"
putexcel F9 = "SYST-EUR"
putexcel F10 = "TOMHS"

import excel "forest_consensus.xlsx", sheet("Table 1") firstrow clear

metan logor selogor if study<=5, eform xlabel(0.5, 1, 1.5, 2, 2.5) random
metan logor selogor if study>=6 & study<=9, eform xlabel(0.5, 1, 1.5, 2, 2.5) random
metan logor selogor, eform xlabel(0.5, 1, 1.5, 2, 2.5) label(namevar=studylabel) xsize(5) ysize(5) random


clear

***********************Systolic****************************************
*Use dataset_dummy.dta

use dataset_dummy.dta, clear


putexcel set "forest_sysdef.xlsx", sheet("Table 1", replace) replace

putexcel A1 = "study"
putexcel B1 = "groups"
putexcel C1 = "number"
putexcel D1 = "logor"
putexcel E1 = "selogor"
putexcel F1 = "studylabel"

local count_row = 2

forvalues s=1/9 {
```

```
xtgee sOHTN_v2 low_goal if vis!=0 & study==`s' & a_group_v2==1, i(newid) family(binomial) link(logit)
robust
matrix b=e(b)
matrix V=e(V)
sca N_g=e(N_g)
sca N_m=e(N)
putexcel A`count_row' = `s'
putexcel B`count_row' = `=scalar(N_g)'
putexcel C`count_row' = `=scalar(N_m)'
lincom low_goal
putexcel D`count_row' = `r(estimate)'
putexcel E`count_row' = `r(se)'

local count_row = `count_row' + 1
}

putexcel A1 = "study"
putexcel F2 = "AASK"
putexcel F3 = "ACCORD"
putexcel F4 = "SPRINT"
putexcel F5 = "SPS3"
putexcel F6 = "UKPDS"
putexcel F7 = "HYVET"
putexcel F8 = "SHEP"
putexcel F9 = "SYST-EUR"
putexcel F10 = "TOMHS"

import excel "forest_sysdef.xlsx", sheet("Table 1") firstrow clear

metan logor selogor if study<=5, eform xlabel(0.5, 1, 1.5, 2, 2.5) random
metan logor selogor if study>=6 & study<=9, eform xlabel(0.5, 1, 1.5, 2, 2.5) random
metan logor selogor, eform xlabel(0.5, 1, 1.5, 2, 2.5) label(namevar=studylabel) xsize(5) ysize(5) random



clear
```

```
********************************************************************************
***********Supplement Table ST5. Means before and after randomization*****************
********************************************************************************
*Annotation: This code was used to generate results included in Supplement Table ST5
*Use dataset_dummy.dta

use dataset_dummy.dta, clear

quietly {
foreach j in sit_sbp_v2 sit_dbp_v2 std_sbp_v2 std_dbp_v2 {
forvalues g=0/1 {
xtgee `j' vis2 if study<=9 & a_group_v2==1 & low_goal==`g', i(newid) family(normal) link(identity) robust
*pre-randomization
lincom _cons
noi di "`j'|" e(N_g) "|" e(N) "|" %4.1f r(estimate) " (" %4.2f r(se) ")"
}
noi di ""
}
}

*post-randomization
quietly {
foreach j in sit_sbp_v2 sit_dbp_v2 std_sbp_v2 std_dbp_v2 {
forvalues g=0/1 {
xtgee `j' vis2 if study<=9 & a_group_v2==1 & low_goal==`g', i(newid) family(normal) link(identity) robust
lincom _cons + vis2
noi di "`j'|" e(N_g) "|" e(N) "|" %4.1f r(estimate) " (" %4.2f r(se) ")"
}
noi di ""
}
}

clear
```

```
********************************************************
***********Supplement Table ST6. Time analysis*****************
********************************************************
*Annotation: This code was used to generate results included in Supplement Table ST6
*Use dataset_dummy.dta

use dataset_dummy.dta, clear

gen study_month=round(time_to_rz/30,1)
gen study_yrs=round(time_to_rz/365.25,1)
gen study_time_cat=0 if study_month<=0 & vis==0 & has_fu_v2==1
replace study_time_cat=1 if vis!=0 & study_month<=1
replace study_time_cat=2 if vis!=0 & study_month>1 & study_month<=6
replace study_time_cat=3 if vis!=0 & study_month>6 & study_month<=12
replace study_time_cat=4 if vis!=0 & study_month>12 & study_month<=24
replace study_time_cat=5 if vis!=0 & study_month>24 & study_month<=36
replace study_time_cat=6 if vis!=0 & study_month>36 & study_month<=48
replace study_time_cat=7 if vis!=0 & study_month>48

gen study_time_cat_med=.
forvalues a=0/7 {
sum study_month if a_group_v2==1 & study_time_cat==`a', d
replace study_time_cat_med=r(p50) if a_group_v2==1 & study_time_cat==`a'
}
tab study_time_cat_med

*Proportions – unadjusted for absolute value, by treatment
quietly {
forvalues j=0/1 {
noi di "low_goal=`j'"
xi: xtgee aOHTN_v2 i.study_time_cat /*i.study*/ if a_group_v2==1 & study<=9 & low_goal==`j', i(newid)
family(binomial) link(logit) robust
matrix b=e(b)
*baseline
lincom _cons
noi di %3.1f 100*exp(r(estimate))/(1+exp(r(estimate)))
*follow-up periods
forvalues i=1/7 {
lincom _cons + _Istudy_tim_`i'
noi di %3.1f 100*exp(r(estimate))/(1+exp(r(estimate)))
}
}
}

*Relative comparisons (ORs; 95% CI)
*Time interaction
quietly {
foreach s in 9 {
```

```
noi di "N trials=`s'"
forvalues a=0/1 {
noi di "low_goal:`a'"
xi: xtgee aOHTN_v2 i.study_time_cat i.study if a_group_v2==1 & study<=`s' & low_goal==`a', i(newid)
family(binomial) link(logit) robust eform
matrix b=e(b)
matrix V=e(V)
forvalues i=1/7 {
noi di e(N_g) "|" e(N) "|" %4.2f exp(b[1,`i']) " (" %-4.2f exp(b[1,`i'] - 1.96*sqrt(V[`i',`i'])) "," %5.2f
exp(b[1,`i'] + 1.96*sqrt(V[`i',`i'])) ")" /*"|" round(2*ttail(e(N), abs(b[1,`i'])/sqrt(V[`i',`i'])),.001)*/
}
}
noi di "p-interaction"
xi: xtgee aOHTN_v2 i.low_goal*i.study_time_cat i.study if a_group_v2==1 & study<=`s', i(newid)
family(binomial) link(logit) robust eform
matrix b=e(b)
matrix V=e(V)
forvalues i=9/15 {
noi di e(N_g) "|" e(N) "|" %4.2f exp(b[1,`i']) " (" %-4.2f exp(b[1,`i'] - 1.96*sqrt(V[`i',`i'])) "," %5.2f
exp(b[1,`i'] + 1.96*sqrt(V[`i',`i'])) ")" "|" round(2*ttail(e(N), abs(b[1,`i'])/sqrt(V[`i',`i'])),.001)
}
}
}

clear
```

```
*********************************************************************
***********Supplement Table 7. Effects on orthostatic change******************
*********************************************************************
*Annotation: This code was used to generate results included in Supplement Table ST7
*Use dataset_dummy.dta

use dataset_dummy.dta, clear

*Continuous difference - BP goal
quietly {
foreach j in diff_sbp_v2 diff_dbp_v2 {
xtgee `j' low_goal i.study if vis!=0 & study<=5 & a_group_v2==1, i(newid) family(normal) link(identity)
robust
matrix b=e(b)
matrix V=e(V)
foreach i in 1 {
noi di "`j'|" e(N_g) "|" e(N) "|" %4.2f (b[1,`i']) " (" %-4.2f (b[1,`i'] - 1.96*sqrt(V[`i',`i'])) "," %5.2f (b[1,`i'] +
1.96*sqrt(V[`i',`i'])) ")" "|" round(2*ttail(e(N), abs(b[1,`i'])/sqrt(V[`i',`i'])),.001)
}
}
}
*Continuous difference - Placebo
quietly {
foreach j in diff_sbp_v2 diff_dbp_v2 {
xtgee `j' low_goal i.study if vis!=0 & study>5 & study<=9 & a_group_v2==1, i(newid) family(normal)
link(identity) robust
matrix b=e(b)
matrix V=e(V)
foreach i in 1 {
noi di "`j'|" e(N_g) "|" e(N) "|" %4.2f (b[1,`i']) " (" %-4.2f (b[1,`i'] - 1.96*sqrt(V[`i',`i'])) "," %5.2f (b[1,`i'] +
1.96*sqrt(V[`i',`i'])) ")" "|" round(2*ttail(e(N), abs(b[1,`i'])/sqrt(V[`i',`i'])),.001)
}
}
}
*Continuous difference - All (extended)
quietly {
foreach j in diff_sbp_v2 diff_dbp_v2 {
xtgee `j' low_goal i.study if vis!=0 & a_group_v2==1, i(newid) family(normal) link(identity) robust
matrix b=e(b)
matrix V=e(V)
foreach i in 1 {
noi di "`j'|" e(N_g) "|" e(N) "|" %4.2f (b[1,`i']) " (" %-4.2f (b[1,`i'] - 1.96*sqrt(V[`i',`i'])) "," %5.2f (b[1,`i'] +
1.96*sqrt(V[`i',`i'])) ")" "|" round(2*ttail(e(N), abs(b[1,`i'])/sqrt(V[`i',`i'])),.001)
}
}
}

clear
```
